# Supplementary material for: DNA 5-methylcytosine detection and methylation phasing using PacBio circular consensus sequencing
Source: Nat Commun. 2023 Jul 8;14:4054. doi: 10.1038/s41467-023-39784-9 (PMC10329642; doi:10.1038/s41467-023-39784-9)
Supplement: Supplementary file 1 — Supplementary infomation [file 41467_2023_39784_MOESM1_ESM.pdf]

Supplementary Information for

DNA 5-methylcytosine detection and methylation phasing using PacBio circular consensus sequencing

Ni *et al.*

Supplementary Figures

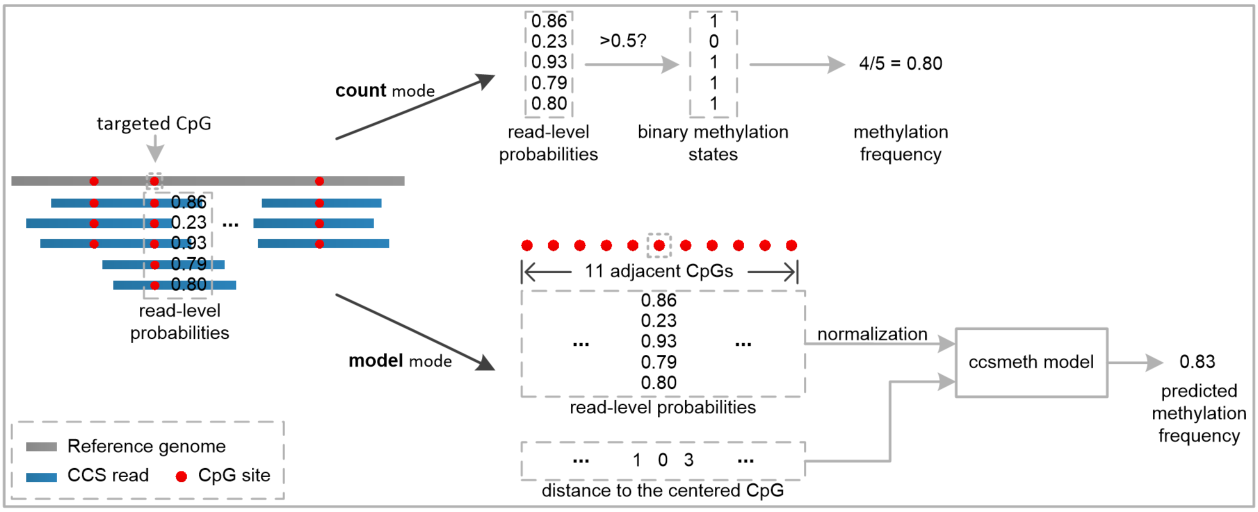

Supplementary Fig. 1 Illustration of inferring methylation frequency of CpGs at site level using count mode and model mode.

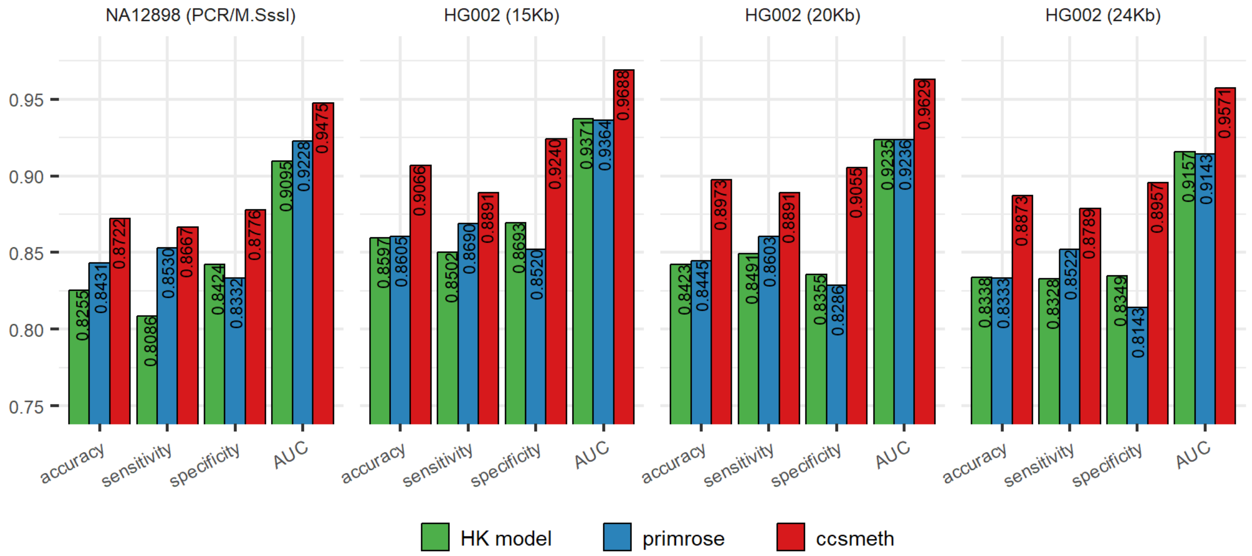

Supplementary Fig. 2 Comparing ccsmeth with HK model and primrose at read level using subsampled 100K reads of NA12898 (10Kb, PCR/M.SssI-treated), HG002 (15Kb, 20Kb, 24Kb). AUC: Area Under the Curve.

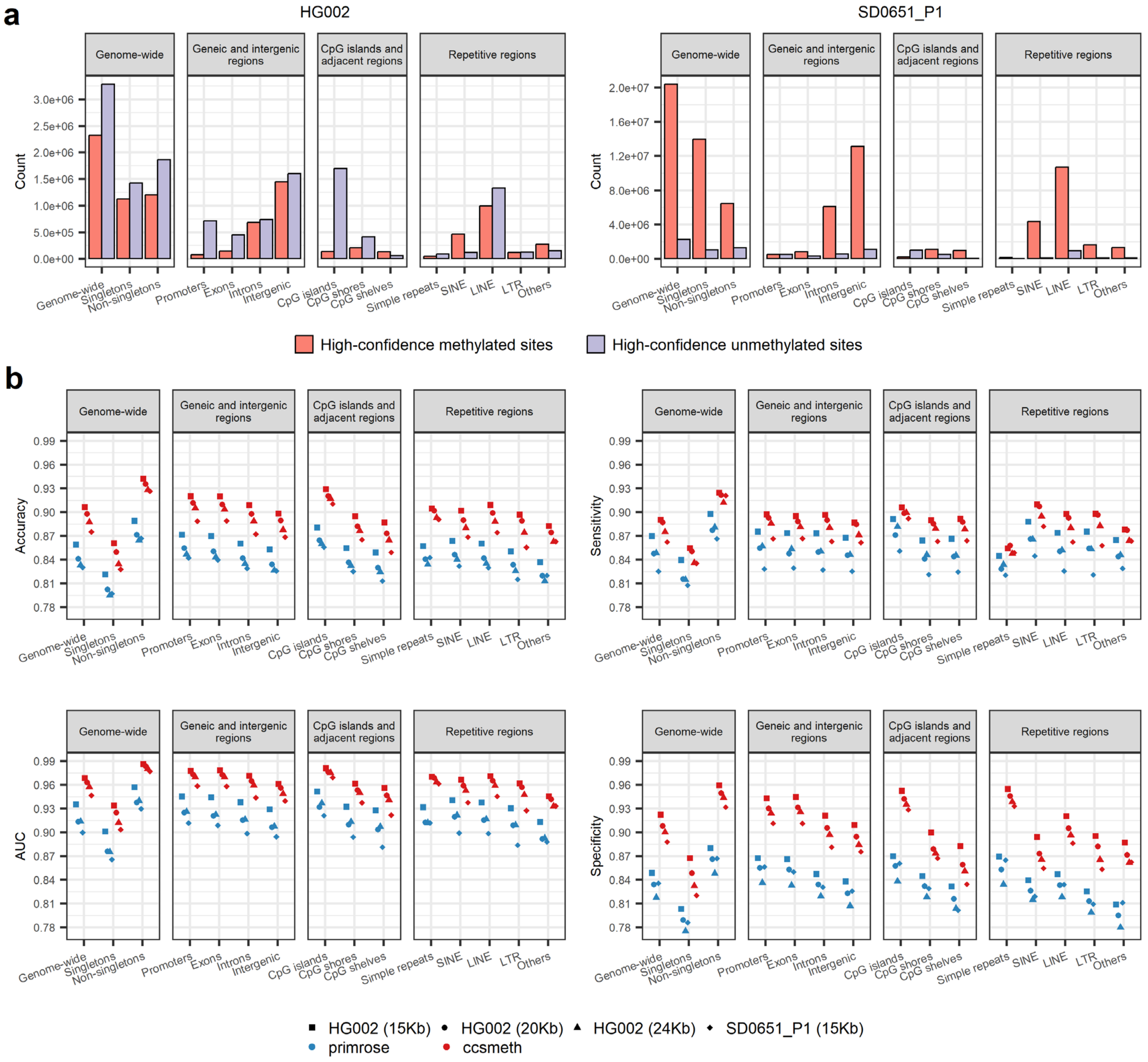

Supplementary Fig. 3 Read-level evaluation of ccsmeth in different genomic contexts and regions. **a** Number of high-confidence methylated and unmethylated sites (CpGs) of HG002 and SD0651_P1 in different genomic contexts and regions. The high-confidence sites are selected based on the results of BS-seq (methylated: coverage ≥ 5 and methylation frequency = 1; unmethylated: coverage ≥ 5 and methylation frequency = 0). **b** Read-level performances of primrose and ccsmeth in different genomic contexts and regions. Source data are provided as a Source Data file.

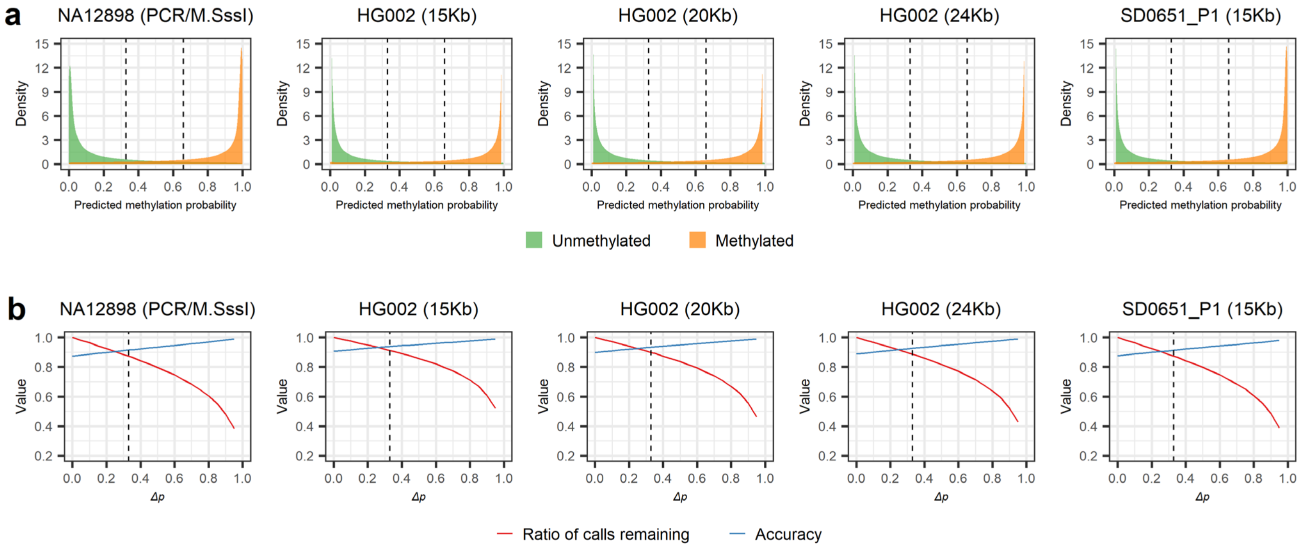

Supplementary Fig. 4 Filtering out ambiguous calls to improve the accuracy of ccsmeth at read level. **a** Distribution of methylation probabilities predicted by ccsmeth for the methylated and unmethylated CpGs. Dash lines in the plots indicate probability = 0.33 and 0.66. **b** Effect of Δ_p on the percentage of remaining calls and the accuracies of ccsmeth for read-level prediction. $\Delta_p = |P_r - P'_r|$, where P_r is methylation probability outputted by ccsmeth for a CpG, $P'_r = 1 - P_r$. Dash lines indicate $\Delta_p = 0.33$. Source data are provided as a Source Data file.

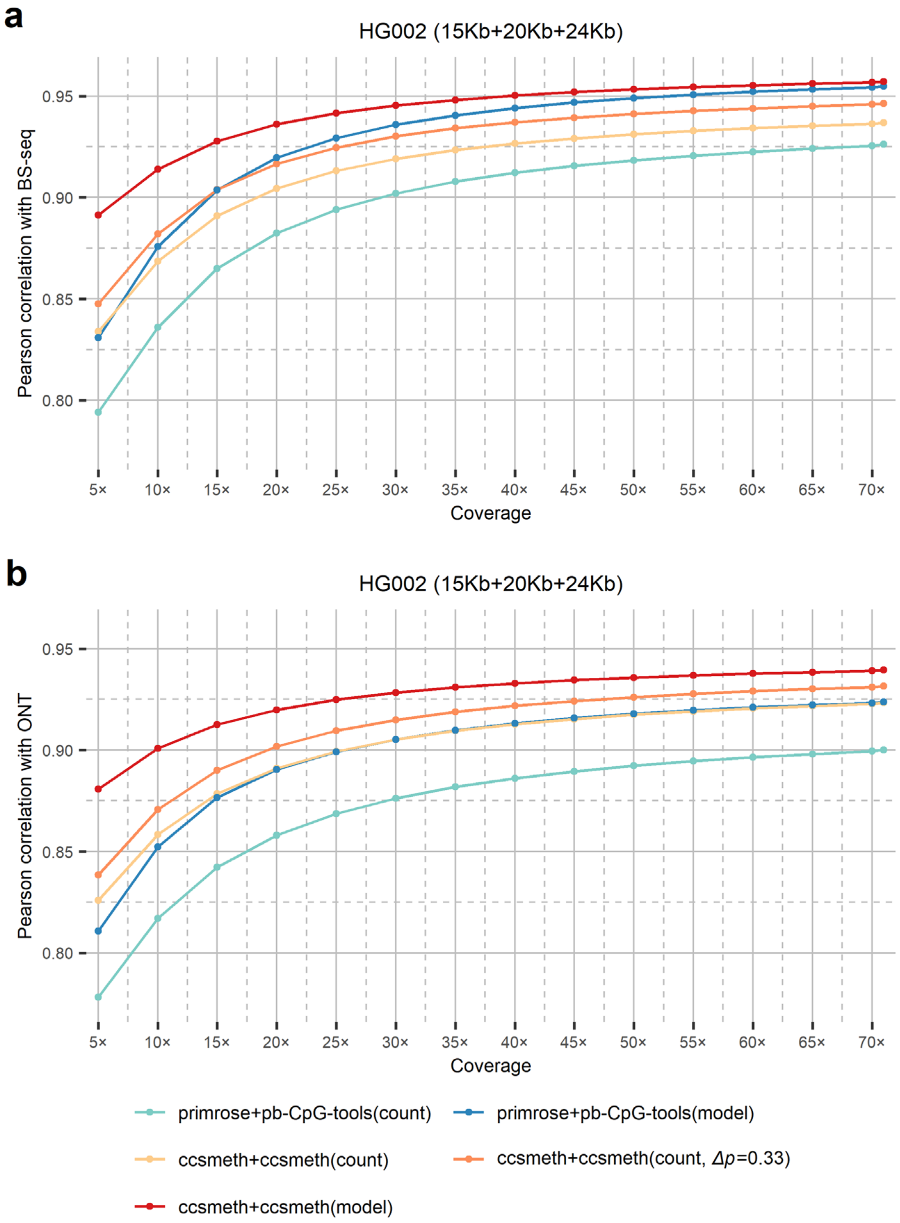

Supplementary Fig. 5 Comparing ccsmeth and primrose/pb-CpG-tools against BS-seq (**a**) and nanopore sequencing (**b**) under different coverages of HG002 CCS reads (71.0× in total). Values for coverage 5×-70× are the average of 5 repeated tests. The standard deviation values of the multiple repeated tests are in Supplementary Data 1. Source data are provided as a Source Data file.

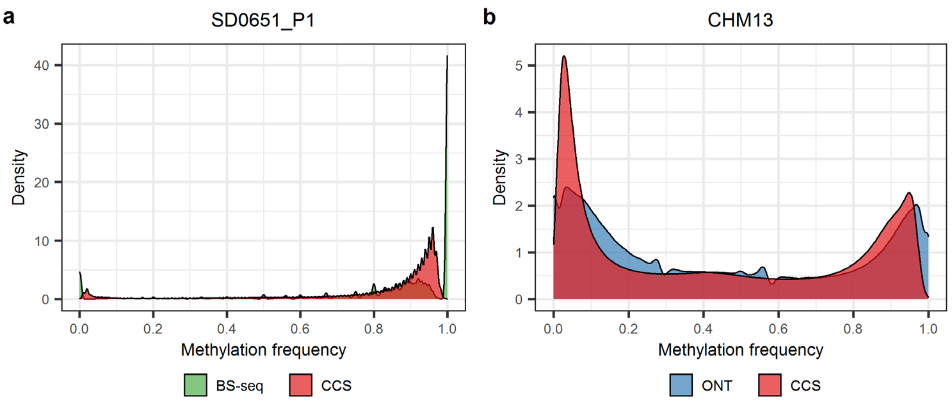

Supplementary Fig. 6 Methylation frequencies of CpGs in two human samples SD0651_P1 and CHM13. **a** Distribution of methylation frequencies of CpGs in SD0651_P1 detected by BS-seq and CCS (ccsmeth in model mode). **b** Distribution of methylation frequencies of CpGs in CHM13 detected by nanopore sequencing and CCS (ccsmeth in model mode). ONT: nanopore sequencing.

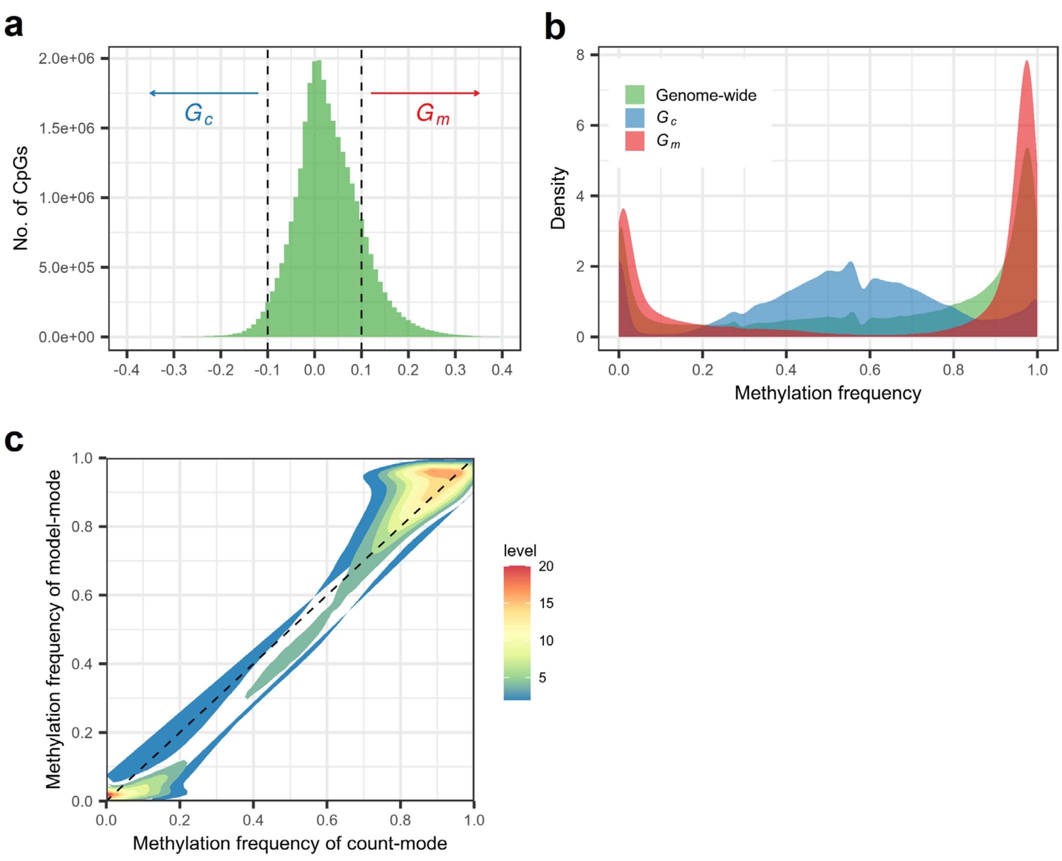

Supplementary Fig. 7 Comparison of the count mode and model mode of ccsmeth using 71.0× HG002 CCS reads. **a** Distribution of the number of CpGs in terms of measuring whether the methylation frequencies of model mode or count mode is closer to that of BS-seq. R_b, R_c, R_m represent the methylation frequencies of a CpG calculated by BS-seq, count mode of ccsmeth, and model mode of ccsmeth, respectively. G_c contains CpGs whose $| R_b - R_c | - | R_b - R_m | < -0.1$, while G_m contains CpGs whose $| R_b - R_c | - | R_b - R_m | > 0.1$. **b** Distribution of the "True" methylation frequencies (calculated by BS-seq) of the CpGs in the whole genome, G_c, and G_m, respectively. **c** Comparison of genome-wide per-site methylation frequency between the count mode and model mode of ccsmeth.

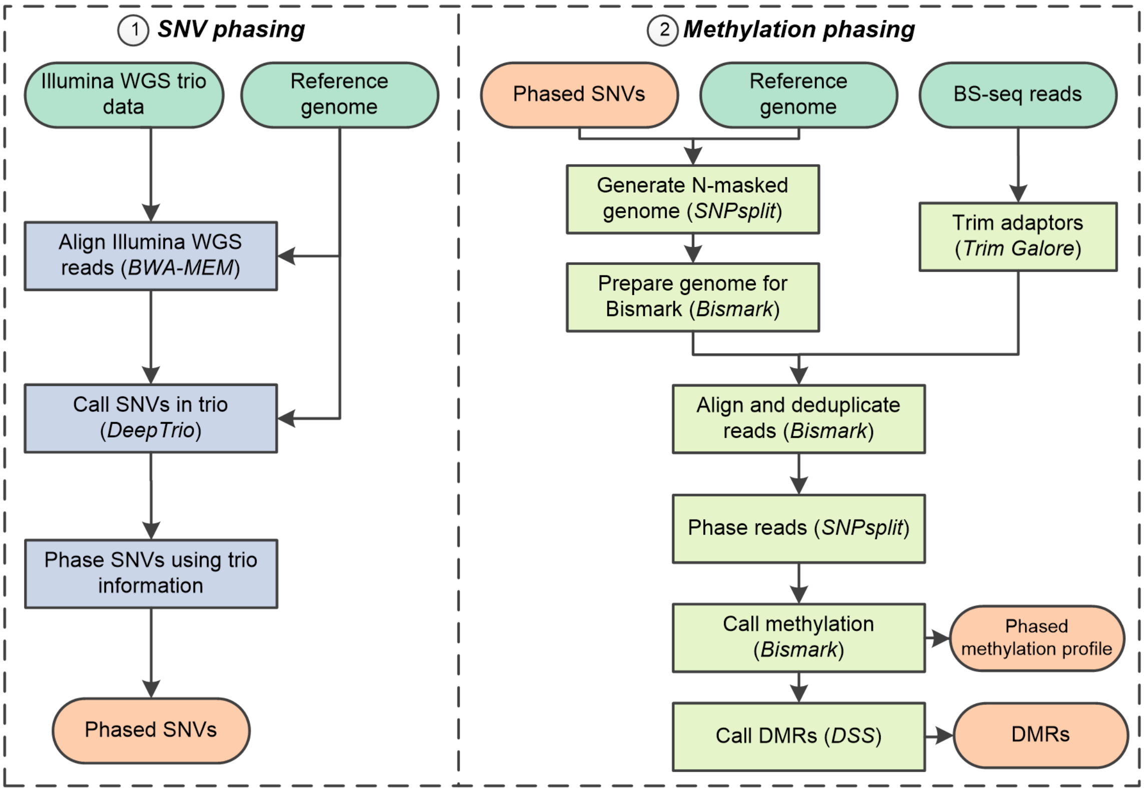

Supplementary Fig. 8 Pipeline of haplotype-aware methylation calling and allele-specific methylation detection using Illumina whole-genome sequencing (WGS) trio data and BS-seq data. SNVs: single nucleotide variants; DMRs: differentially methylated regions.

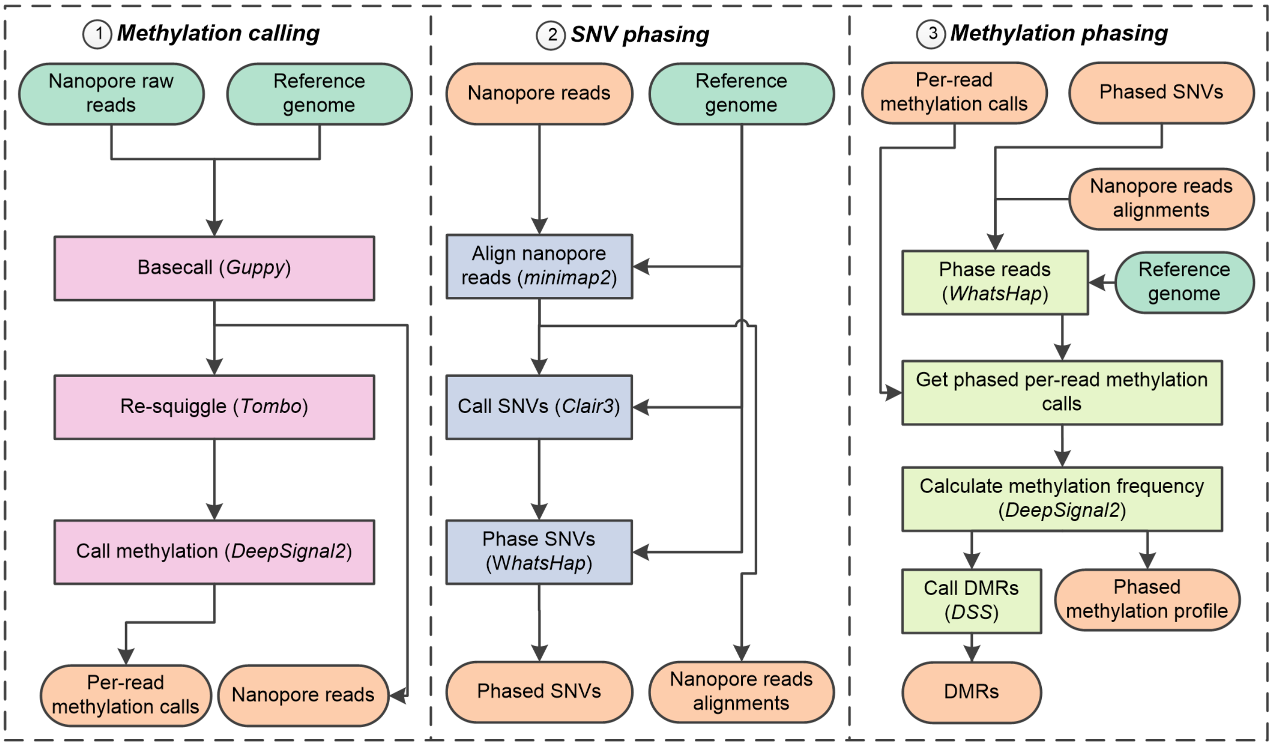

Supplementary Fig. 9 Pipeline of haplotype-aware methylation calling and allele-specific methylation using nanopore data only. SNVs: single nucleotide variants; DMRs: differentially methylated regions.

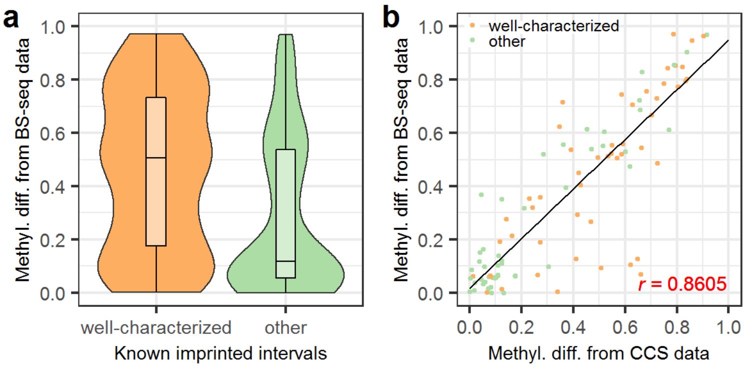

Supplementary Fig. 10 Methylation differences of known imprinted intervals calculated using BS-seq/CCS data in two haplotypes of HG002. **a** Distribution of methylation differences of known imprinted intervals between two haplotypes of HG002 calculated using BS-seq data. 52 out of 102 well-characterized intervals and 46 out of 102 other intervals which have at least 5 CpGs covered by BS-seq reads in each haplotype are analyzed. The boxes inside the violin plots indicate 50th percentile (middle line), 25th and 75th percentile (box), the smallest value within 1.5 times interquatile range below 25th percentile and largest value within 1.5 times interquatile range above 75th percentile (whiskers). **b** Comparison of methylation differences of known imprinted intervals calculated using CCS and BS-seq data. 97 known imprinted intervals which can be covered by BS-seq and CCS data were analyzed. Methyl. diff.: methylation difference; *r*: Pearson correlation. Source data are provided as a Source Data file.

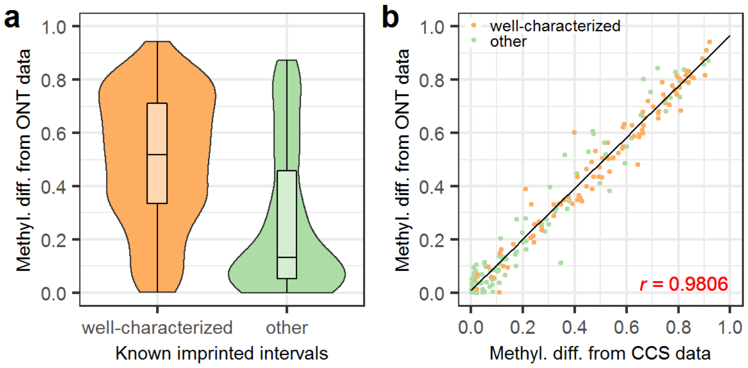

Supplementary Fig. 11 Methylation differences of known imprinted intervals calculated using nanopore/CCS data in two haplotypes of HG002. **a** Distribution of methylation differences of known imprinted intervals between two haplotypes of HG002 calculated using nanopore data. 98 out of 102 well-characterized intervals and 96 out of 102 other intervals which have at least 5 CpGs covered by nanopore reads in each haplotype are analyzed. The boxes inside the violin plots indicate 50th percentile (middle line), 25th and 75th percentile (box), the smallest value within 1.5 times interquatile range below 25th percentile and largest value within 1.5 times interquatile range above 75th percentile (whiskers). **b** Comparison of methylation differences of known imprinted intervals calculated using CCS and nanopore data. 191 known imprinted intervals which can be covered by nanopore and CCS data were analyzed. Methyl. diff.: methylation difference; ONT: nanopore sequencing; *r*: Pearson correlation. Source data are provided as a Source Data file.

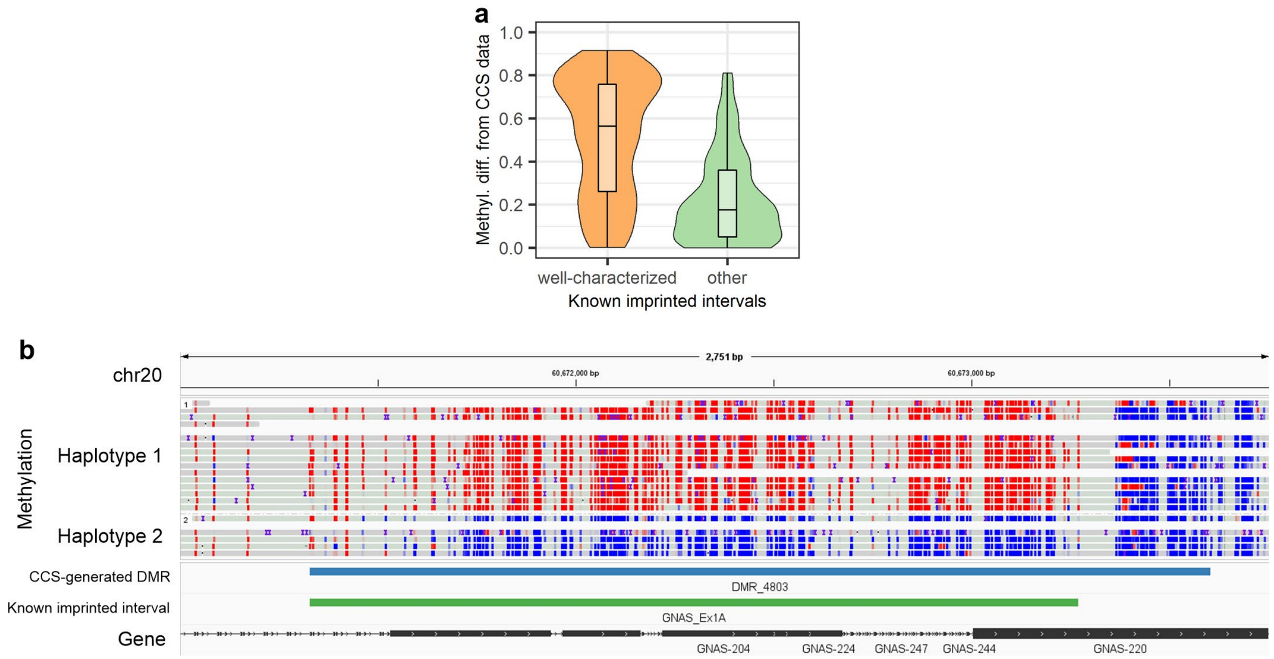

Supplementary Fig. 12 Methylation phasing of ccsmethphase on SD0651_P1 CCS data. **a** Distribution of methylation differences of known imprinted intervals calculated using CCS data between two haplotypes of SD0651_P1. 93 out of 102 "well-characterized" intervals, and 91 out of 102 "other" intervals which have at least 5 CpGs covered by CCS reads in each haplotype are analyzed. The boxes inside the violin plots indicate 50th percentile (middle line), 25th and 75th percentile (box), the smallest value within 1.5 times interquatile range below 25th percentile and largest value within 1.5 times interquatile range above 75th percentile (whiskers). Source data are provided as a Source Data file. **b** Screenshot of Integrative Genomics Viewer (chr20:60,671,001-60,673,750) on a DMR of SD0651_P1 near the maternally imprinted gene *GNAS*. Red and blue dots represent CpGs with high and low methylation probabilities, respectively.

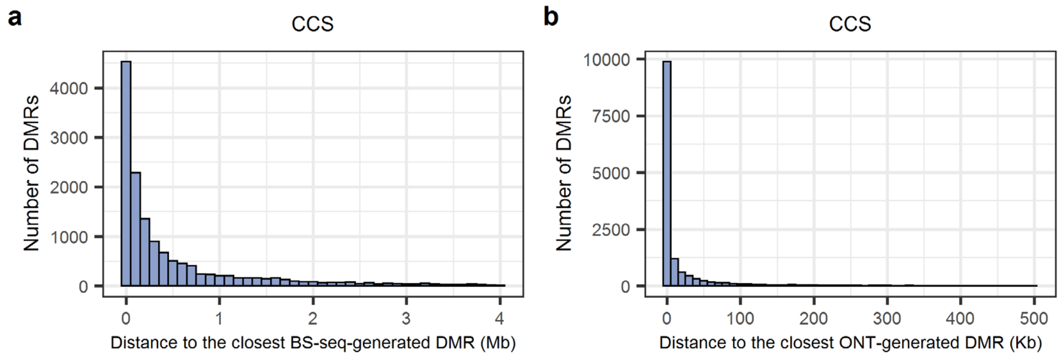

Supplementary Fig. 13 Distribution of the number of CCS-generated DMRs in terms of distance to the closest BS-seq-generated (**a**) and ONT-generated DMR (**b**) in HG002. ONT: nanopore sequencing. Source data are provided as a Source Data file.

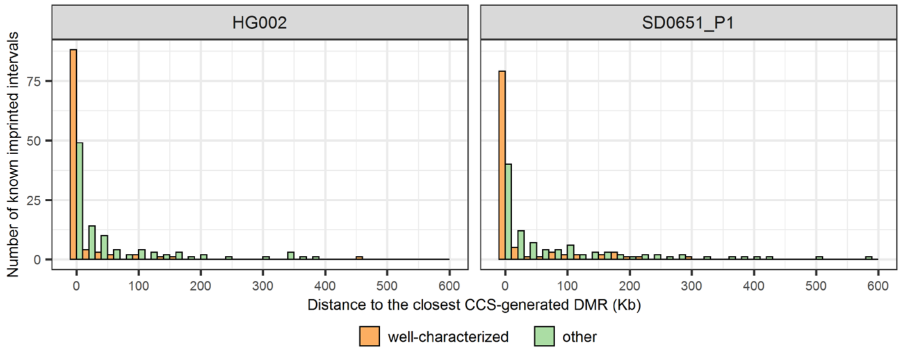

Supplementary Fig. 14 Distribution of the number of known imprinted intervals in terms of distance to the closest CCS-generated DMR in HG002 and SD0651_P1. Source data are provided as a Source Data file.

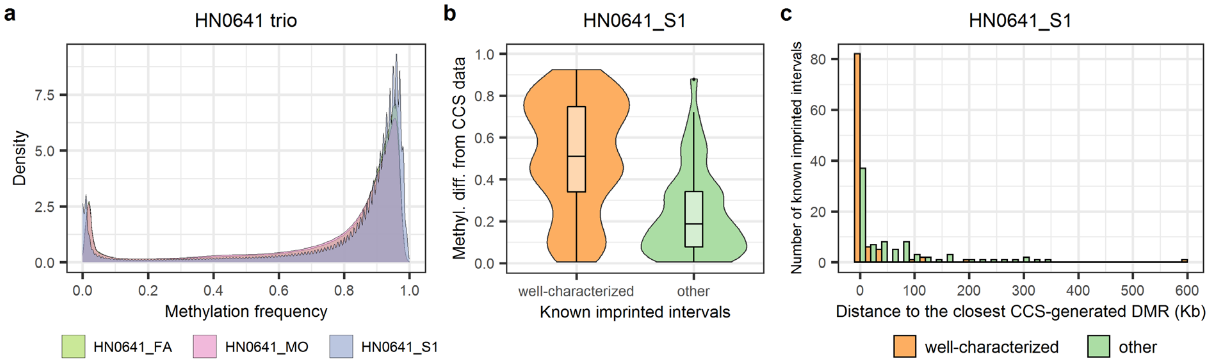

Supplementary Fig. 15 5mCpG detection and methylation phasing of the HN0641 family trio using ccsmethphase. **a** Distribution of methylation frequencies of CpGs in HN0641_FA (father), HN0641_MO (mother), and HN0641_S1 (son). **b** Distribution of methylation differences of known imprinted intervals between two haplotypes of HN0641_S1. 93 out of 102 "well-characterized" intervals, and 93 out of 102 "other" intervals which have at least 5 CpGs covered by CCS reads in each haplotype are analyzed. The boxes inside the violin plots indicate 50th percentile (middle line), 25th and 75th percentile (box), the smallest value within 1.5 times interquatile range below 25th percentile and largest value within 1.5 times interquatile range above 75th percentile (whiskers). **c** Distribution of the number of known imprinted intervals in terms of distance to the closest CCS-generated DMR in HN0641_S1. Source data underlying **b** and **c** are provided as a Source Data file.

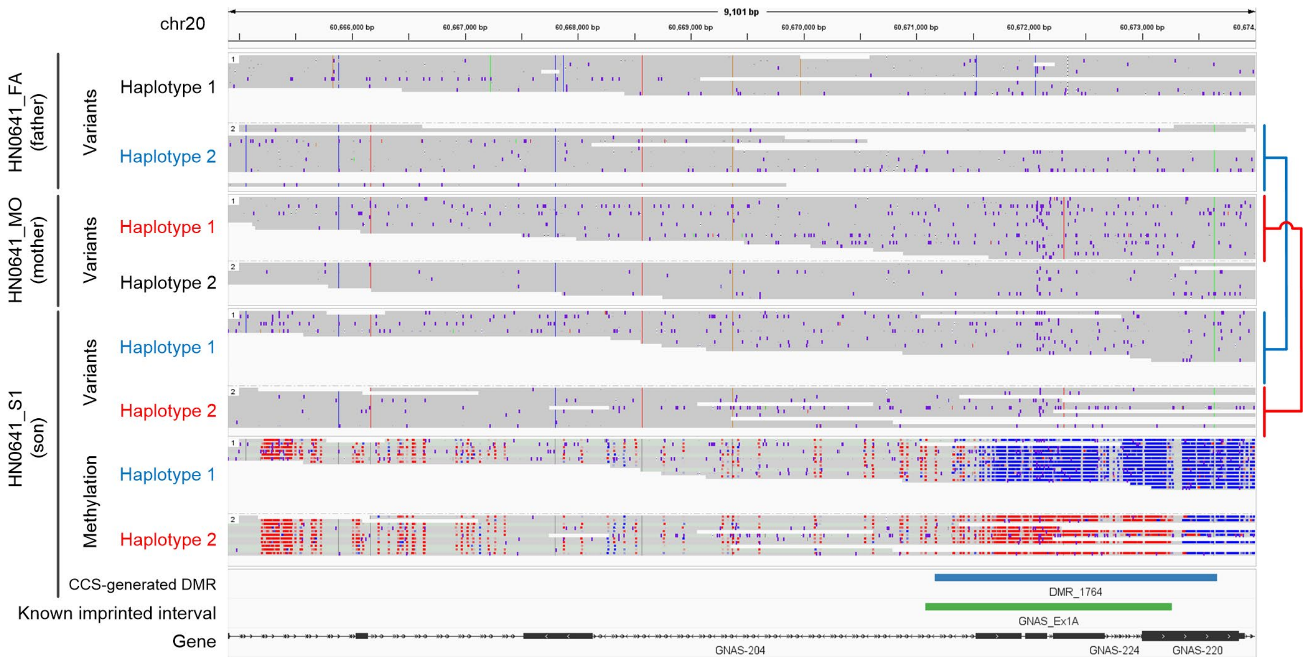

Supplementary Fig. 16 Screenshot of Integrative Genomics Viewer[1] (chr20:60,664,900-60,673,999) on a DMR of HN0641_S1 near the **maternally** imprinted gene *GNAS*, showing the variants information of the HN0641 family trio, and the phased methylation information of HN0641_S1. Red and blue dots in the "Methylation" area represent CpGs with high and low methylation probabilities, respectively.

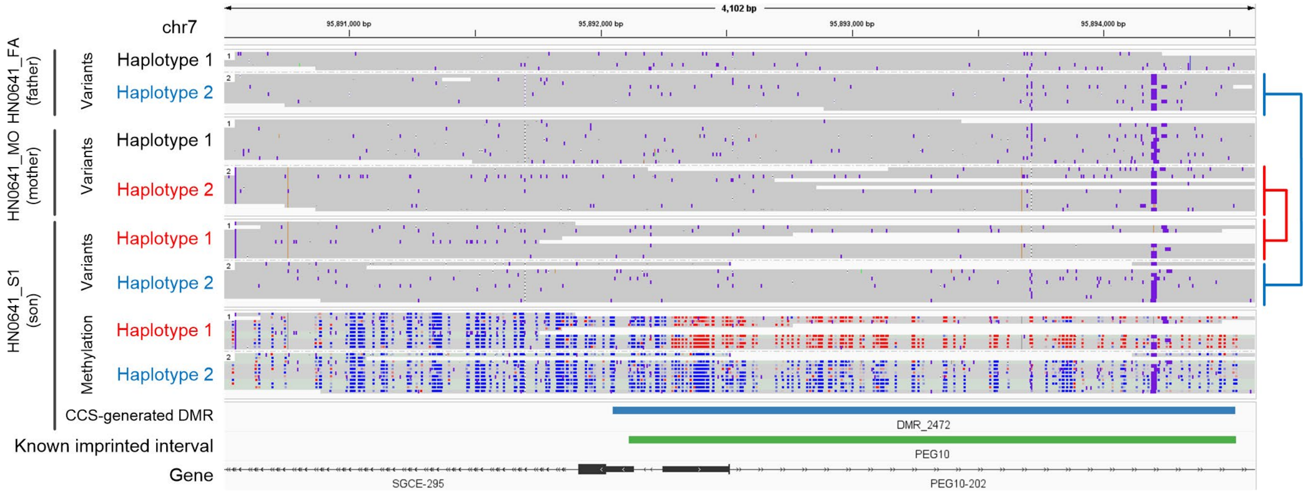

Supplementary Fig. 17 Screenshot of Integrative Genomics Viewer (chr7:95,890,500-95,894,600) on a DMR of HN0641_S1 near the **maternally** imprinted gene *PEG10*, showing the variants information of the HN0641 family trio, and the phased methylation information of HN0641_S1. Red and blue dots in the "Methylation" area represent CpGs with high and low methylation probabilities, respectively.

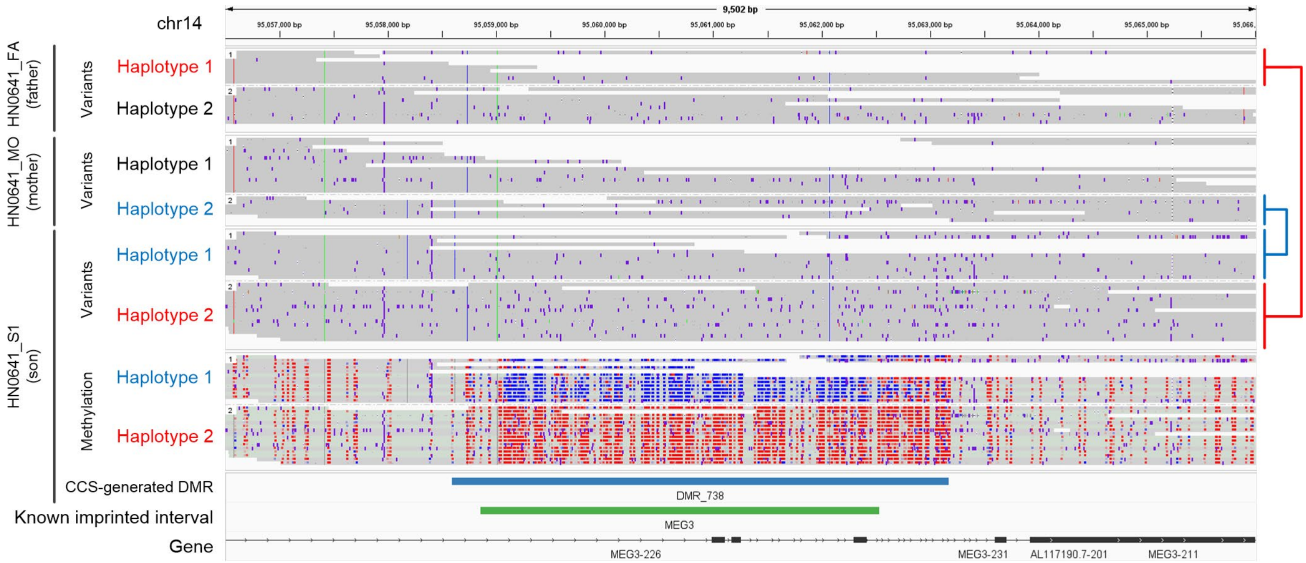

Supplementary Fig. 18 Screenshot of Integrative Genomics Viewer (chr14:95,056,500-95,066,00) on a DMR of HN0641_S1 near the **paternally** imprinted gene *MEG3*, showing the variants information of the HN0641 family trio, and the phased methylation information of HN0641_S1. Red and blue dots in the "Methylation" area represent CpGs with high and low methylation probabilities, respectively.

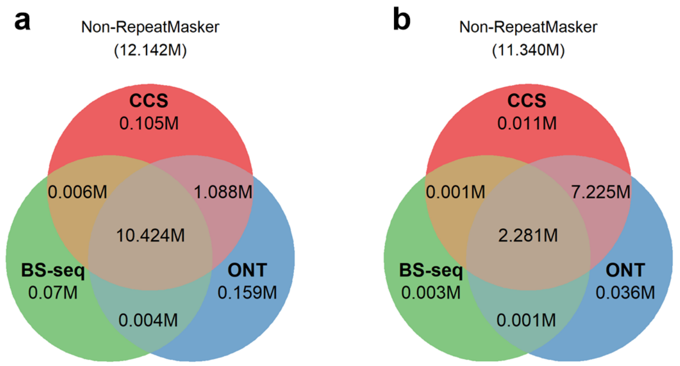

Supplementary Fig. 19 Comparison of the number of total (**a**) and phased (**b**) CpGs detected by the HG002 BS-seq (117.5×), ONT (65.8×), and CCS (71.0×) reads in non-RepeatMasker regions.

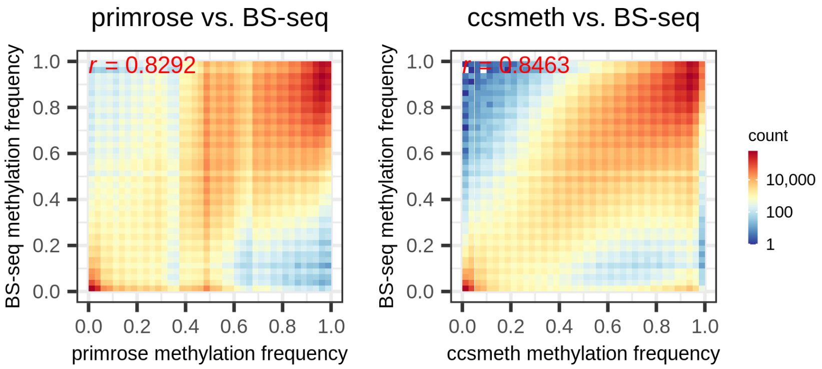

Supplementary Fig. 20 Comparing ccsmeth and primrose with BS-seq for 5mCpG detection of a Zebrafish sample.

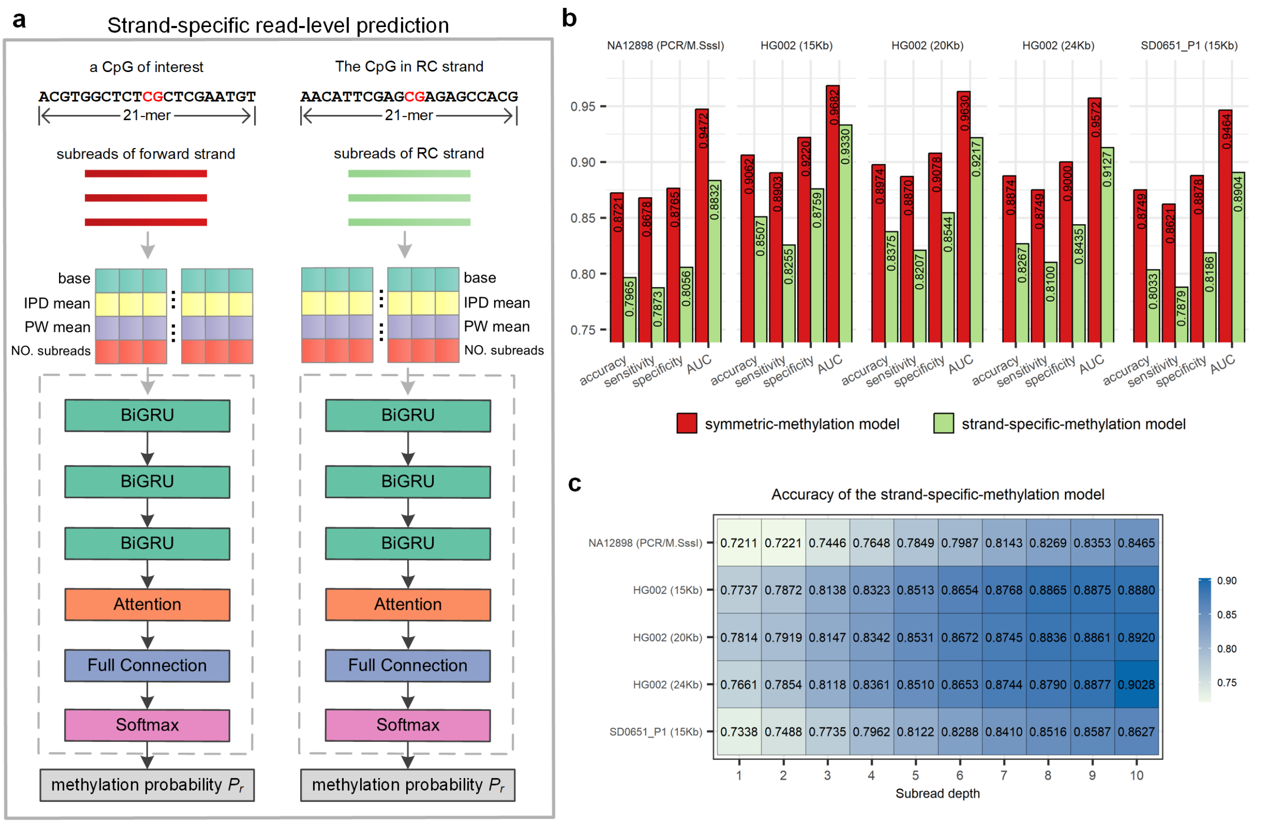

Supplementary Fig. 21 ccsmeth for strand-specific methylation detection. **a** The model framework of ccsmeth for strand-specific methylation detection. **b** Comparison of the strand-specific-methylation model and the symmetric-methylation model of ccsmeth at read-level 5mCpG detection using long CCS reads. **c** Accuracy of the strand-specific-methylation model under different subread depths.

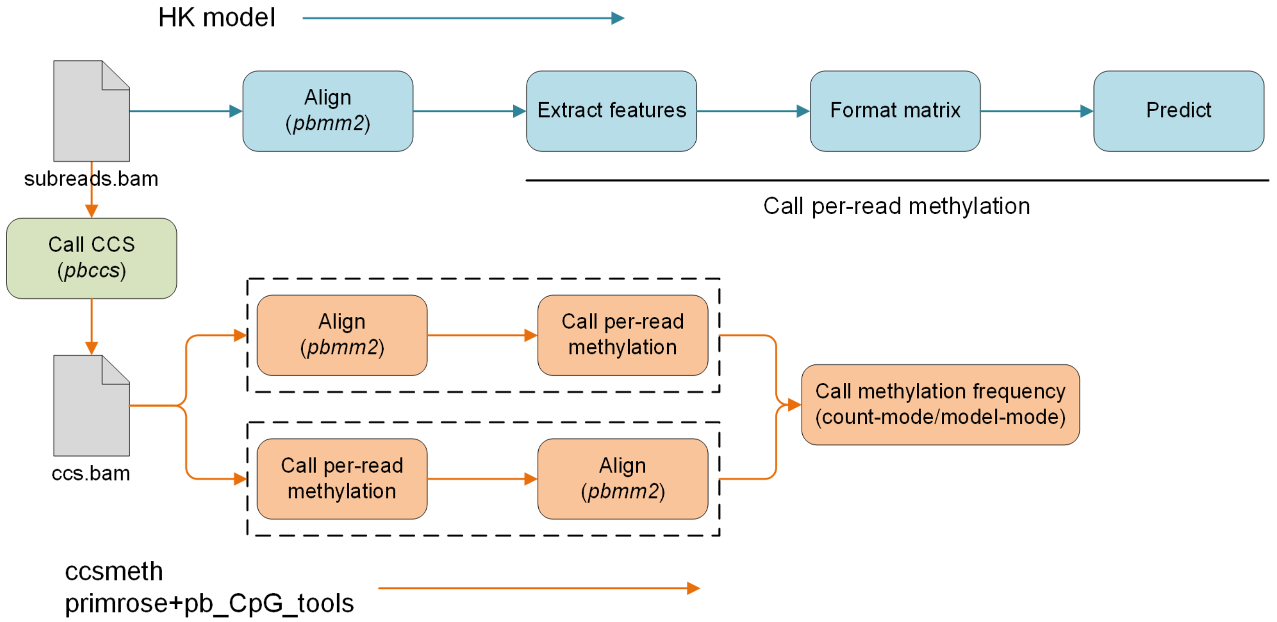

Supplementary Fig. 22 Main steps of HK model, ccsmeth, and primrose for methylation calling.

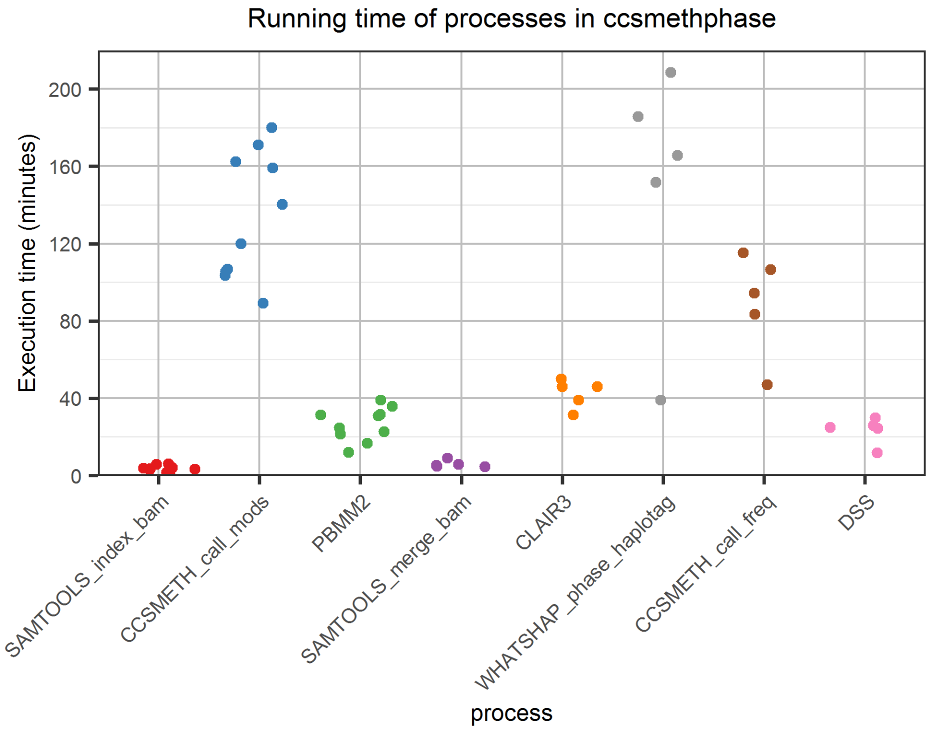

Supplementary Fig. 23 Runtime of 8 main processes in ccsmethphase. 10 SMRT cells of CCS reads (2 SMRT cells for each of the 5 "samples": HG002 (15Kb), HG002 (20Kb), HG002 (24Kb), CHM13 (20Kb), and SD0651_P1 (15Kb)) were used in this test. Source data are provided as a Source Data file.

Supplementary Tables

Supplementary Table 1 Statistics of PacBio CCS datasets used in this study. read depth: the number of passed subreads for each CCS read; HPRC: Human Pangenome Reference Consortium.

sample	cell ID	DNA material	sequencing kit	insert size	NO. of CCS reads	mean read length	mean read depth	source
M01	m54276_180627_125201	M.SssI-treated	sequel I kit 3.0	-	423,470	440.54	42.44	Tse. *et al.*[2]
W01	m54276_180627_023725	PCR-treated	sequel I kit 3.0	-	444,310	468.12	41.66	Tse. *et al.*[2]
M02	m64042_190713_204343	M.SssI-treated	sequel II kit 1.0	-	1,144,141	4,905.04	21.86	Tse. *et al.*[2]
W02	m64042_190712_093601	PCR-treated	sequel II kit 1.0	-	1,476,534	6,299.35	19.23	Tse. *et al.*[2]
M03	m64095_200324_133820	M.SssI-treated	sequel II kit 2.0	-	96,933	506.72	52.85	Tse. *et al.*[2]
W03	m64095_200321_184826	PCR-treated	sequel II kit 2.0	-	170,347	894.64	49.41	Tse. *et al.*[2]
NA12898	m64173_220705_133926	PCR/M.SssI-treated	sequel II kit 2.0	10Kb	2,018,018	8,670.84	14.12	in house
HG002	m64012_190921_234837	native	sequel II kit 2.0	15Kb	2,389,655	12,871.53	11.09	HPRC[3]
	m64012_190920_173625	native	sequel II kit 2.0	15Kb	2,349,370	12,867.23	11.05	HPRC[3]
	m64015_190920_185703	native	sequel II kit 2.0	15Kb	2,266,781	12,875.45	10.97	HPRC[3]
	m64008_201124_002822	native	sequel II kit 2.0	15Kb	2,689,222	15,084.49	11.29	Baid *et al.*[4]
	m64194_201120_222723	native	sequel II kit 2.0	15Kb	2,606,147	15,213.73	11.05	Baid *et al.*[4]
	m64011_190830_220126	native	sequel II kit 2.0	20Kb	1,472,376	18,521.25	9.04	HPRC[3]
	m64011_190901_095311	native	sequel II kit 2.0	20Kb	1,395,877	18,516.36	9.00	HPRC[3]
	m64014_200920_132517	native	sequel II kit 2.0	24Kb	1,919,428	24,160.38	7.84	Baid *et al.*[4]
	m64179e_200919_061936	native	sequel II kit 2.0	24Kb	1,742,401	24,437.28	7.60	Baid *et al.*[4]
SD0651_P1	m64114_211125_095059	native	sequel II kit 2.0	15Kb	1,641,522	15,967.21	10.37	in house
	m64242e_211129_171024	native	sequel II kit 2.0	15Kb	2,178,549	16,211.05	11.07	in house
CHM13	m64062_190803_042216	native	sequel II kit 2.0	20Kb	1,433,166	20,760.04	7.72	Nurk *et al.*[5]
	m64062_190806_063919	native	sequel II kit 2.0	20Kb	1,045,868	20,762.52	7.80	Nurk *et al.*[5]
HN0641_FA	m64242e_211230_172120	native	sequel II kit 2.0	15Kb	2,105,955	15,010.29	10.71	in house
	m64053_220125_054827	native	sequel II kit 2.0	15Kb	2,379,680	15,359.23	12.14	in house
HN0641_MO	m64242e_220101_041819	native	sequel II kit 2.0	15Kb	2,198,341	16,179.39	10.60	in house
	m64053_220126_152530	native	sequel II kit 2.0	15Kb	2,120,743	16,363.45	11.16	in house
HN0641_S1	m64242e_220102_151613	native	sequel II kit 2.0	15Kb	1,851,159	16,035.47	10.15	in house
	m64116_220101_042359	native	sequel II kit 2.0	15Kb	2,254,452	16,107.67	10.82	in house

Supplementary Table 2 Partition of PacBio long (≥10Kb) CCS reads to evaluate ccsmeth. $training_1$: Datasets used to train the model of ccsmeth for read-level 5mCpG prediction; $training_2$: Datasets used to train ccsmeth for site-level 5mCpG prediction.

partition	sample	cell ID	insert size	chromosomes	evaluation
$training_1$	NA12898	m64173_220705_133926	10Kb	chr1-22	-
	HG002	m64012_190921_234837	15Kb	chr1-22, chrX, chrY	-
$training_2$	HG002	m64012_190920_173625	15Kb	chr1-22, chrX, chrY	-
		m64015_190920_185703	15Kb	chr1-22, chrX, chrY	-
testing	NA12898	m64173_220705_133926	10Kb	chrX	read-level
	HG002	m64008_201124_002822	15Kb	chr1-22, chrX, chrY	read-level, site-level
		m64194_201120_222723	15Kb	chr1-22, chrX, chrY	read-level, site-level
		m64011_190830_220126	20Kb	chr1-22, chrX, chrY	read-level, site-level
		m64011_190901_095311	20Kb	chr1-22, chrX, chrY	read-level, site-level
		m64014_200920_132517	24Kb	chr1-22, chrX, chrY	read-level, site-level
		m64179e_200919_061936	24Kb	chr1-22, chrX, chrY	read-level, site-level
	SD0651_P1	m64114_211125_095059	15Kb	chr1-22, chrX, chrY	read-level, site-level
		m64242e_211129_171024	15Kb	chr1-22, chrX, chrY	read-level, site-level
	CHM13	m64062_190803_042216	20Kb	chr1-22, chrX	site-level
		m64062_190806_063919	20Kb	chr1-22, chrX	site-level
	HN0641_FA	m64242e_211230_172120	15Kb	chr1-22, chrX, chrY	ASM detection
		m64053_220125_054827	15Kb	chr1-22, chrX, chrY	ASM detection
	HN0641_MO	m64242e_220101_041819	15Kb	chr1-22, chrX, chrY	ASM detection
		m64053_220126_152530	15Kb	chr1-22, chrX, chrY	ASM detection
	HN0641_S1	m64242e_220102_151613	15Kb	chr1-22, chrX, chrY	ASM detection
		m64116_220101_042359	15Kb	chr1-22, chrX, chrY	ASM detection

Supplementary Table 3 Illumina and nanopore datasets used in this study. ONT: Oxford Nanopore Technologies; GIAB: Genome in a Bottle.

sample	type	(mean) read length	mean genome coverage	source
HG002	Illumina BS-seq	2×150	117.5×	ONT[6]
	ONT R9.4.1	21,933	65.8×	ONT[6]
	Illumina WGS	2×250	63.1×	GIAB[7]
HG003	Illumina WGS	2×250	55.7×	GIAB[7]
HG004	Illumina WGS	2×250	67.9×	GIAB[7]
SD0651_P1	Illumina BS-seq	2×150	15.7×	in house
CHM13	ONT R9.4.1	19,891	41.8×	Nurk *et al.*[5]

Supplementary Table 4 Evaluation of ccsmeth on 5mCpG detection at read level. Values in the table are the average and standard deviation of 5 repeated tests in "average±std" format.

dataset	method	accuracy	sensitivity	specificity	AUC
M01&W01	HK model	0.8696±0.0006	0.8443±0.0008	0.8950±0.0007	0.9440±0.0003
	ccsmeth	0.9232±0.0004	0.9326±0.0005	0.9137±0.0005	0.9767±0.0001
M02&W02	HK model	0.8346±0.0009	0.8152±0.0009	0.8541±0.0012	0.9156±0.0005
	ccsmeth	0.8788±0.0005	0.8744±0.0005	0.8833±0.0008	0.9496±0.0003
M03&W03	HK model	0.8395±0.0011	0.7839±0.0013	0.8951±0.0012	0.9202±0.0007
	ccsmeth	0.8765±0.0005	0.8541±0.0008	0.8988±0.0011	0.9465±0.0003
NA12898 (pcr/M.SssI)	primrose	0.8432±0.0005	0.8530±0.0008	0.8333±0.0006	0.9230±0.0002
	ccsmeth	0.8721±0.0004	0.8678±0.0004	0.8765±0.0007	0.9472±0.0003
HG002 (15kb)	primrose	0.8590±0.0002	0.8695±0.0007	0.8485±0.0007	0.9350±0.0003
	ccsmeth	0.9062±0.0006	0.8903±0.0013	0.9220±0.0007	0.9682±0.0002
HG002 (20kb)	primrose	0.8408±0.0005	0.8476±0.0013	0.8340±0.0013	0.9131±0.0006
	ccsmeth	0.8974±0.0004	0.8870±0.0004	0.9078±0.0006	0.9630±0.0004
HG002 (24kb)	primrose	0.8330±0.0007	0.8483±0.0008	0.8177±0.0010	0.9137±0.0008
	ccsmeth	0.8874±0.0005	0.8749±0.0007	0.9000±0.0007	0.9572±0.0002
SD0651_P1 (15Kb)	primrose	0.8304±0.0007	0.8251±0.0006	0.8357±0.0013	0.8998±0.0004
	ccsmeth	0.8749±0.0003	0.8621±0.0005	0.8878±0.0006	0.9464±0.0003

Supplementary Table 5 Evaluation of ccsmeth and primrose at genome-wide site level against **BS-seq** on **HG002 (15Kb)** dataset. We compared CpGs covered by at least 5 reads in both CCS and BS-seq datasets. For coverage 5×-25×, we subsampled corresponding coverage reads from the total reads, and repeated the subsampling 5 times. Values in the table for coverage 5×-25× are the average and standard deviation of 5 repeated tests in "average±std" format. *r*: Pearson correlation; r^2: the coefficient of determination; ρ: Spearman correlation; RMSE: root mean square error.

coverage	method		r	r^2	ρ	RMSE
	read-level calling	site-level calling				
5×	primrose	pb-CpG-tools (count)	0.8145±0.0002	0.6635±0.0003	0.7454±0.0004	0.2058±0.0001
	ccsmeth	ccsmeth (count)	0.8478±0.0002	0.7187±0.0003	0.7741±0.0003	0.1907±0.0001
	ccsmeth	ccsmeth (count, Δ_p=0.33)	0.8605±0.0002	0.7404±0.0003	0.7851±0.0003	0.1866±0.0001
	primrose	pb-CpG-tools (model)	0.8449±0.0001	0.7139±0.0002	0.7785±0.0003	0.21±0.0001
	primrose	ccsmeth (model)	0.8784±0.0002	0.7715±0.0003	0.8314±0.0002	0.1694±0.0001
	ccsmeth	ccsmeth (model)	0.8993±0.0001	0.8088±0.0002	0.851±0.0002	0.1579±0.0001
10×	primrose	pb-CpG-tools (count)	0.853±0.0001	0.7277±0.0001	0.7821±0.0001	0.1854±0
	ccsmeth	ccsmeth (count)	0.8798±0.0001	0.7741±0.0001	0.805±0.0001	0.1702±0
	ccsmeth	ccsmeth (count, Δ_p=0.33)	0.8919±0.0001	0.7956±0.0001	0.8145±0.0001	0.1628±0
	primrose	pb-CpG-tools (model)	0.8864±0.0001	0.7857±0.0001	0.8192±0.0001	0.1781±0
	primrose	ccsmeth (model)	0.9023±0.0001	0.8142±0.0001	0.8554±0.0001	0.1542±0
	ccsmeth	ccsmeth (model)	0.9198±0.0001	0.8459±0.0001	0.872±0	0.1433±0.0001
15×	primrose	pb-CpG-tools (count)	0.8793±0	0.7732±0.0001	0.8083±0	0.171±0
	ccsmeth	ccsmeth (count)	0.9008±0	0.8114±0.0001	0.827±0	0.1559±0
	ccsmeth	ccsmeth (count, Δ_p=0.33)	0.9121±0	0.8319±0.0001	0.836±0	0.1463±0
	primrose	pb-CpG-tools (model)	0.9122±0	0.8321±0	0.8462±0	0.1542±0
	primrose	ccsmeth (model)	0.9176±0.0001	0.8419±0.0001	0.872±0	0.1429±0
	ccsmeth	ccsmeth (model)	0.9326±0.0001	0.8697±0.0001	0.8866±0	0.1327±0.0001
20×	primrose	pb-CpG-tools (count)	0.8953±0	0.8015±0	0.8249±0	0.1622±0
	ccsmeth	ccsmeth (count)	0.9133±0	0.8341±0	0.841±0	0.1472±0
	ccsmeth	ccsmeth (count, Δ_p=0.33)	0.9238±0	0.8534±0	0.8499±0	0.1362±0
	primrose	pb-CpG-tools (model)	0.9265±0	0.8585±0	0.8624±0	0.1394±0
	primrose	ccsmeth (model)	0.9269±0	0.8591±0	0.8827±0	0.1353±0
	ccsmeth	ccsmeth (model)	0.9403±0	0.8841±0	0.896±0	0.1258±0
25×	primrose	pb-CpG-tools (count)	0.9056±0	0.8202±0	0.836±0	0.1565±0
	ccsmeth	ccsmeth (count)	0.9213±0	0.8489±0	0.8504±0	0.1416±0
	ccsmeth	ccsmeth (count, Δ_p=0.33)	0.9313±0	0.8673±0	0.8593±0	0.1296±0
	primrose	pb-CpG-tools (model)	0.9354±0	0.8749±0	0.8739±0	0.1297±0
	primrose	ccsmeth (model)	0.933±0	0.8705±0.0001	0.8899±0	0.1301±0
	ccsmeth	ccsmeth (model)	0.9453±0	0.8935±0	0.9022±0	0.121±0
25.6×(all)	primrose	pb-CpG-tools (count)	0.9077	0.8240	0.8383	0.1554
	ccsmeth	ccsmeth (count)	0.9229	0.8518	0.8523	0.1404
	ccsmeth	ccsmeth (count, Δ_p=0.33)	0.9328	0.87	0.8612	0.1283
	primrose	pb-CpG-tools (model)	0.9371	0.8782	0.8764	0.1278
	primrose	ccsmeth (model)	0.9342	0.8728	0.8913	0.1290
	ccsmeth	ccsmeth (model)	0.9463	0.8955	0.9035	0.1201

Supplementary Table 6 Evaluation of ccsmeth and primrose at genome-wide site level against **nanopore sequencing** on **HG002 (15Kb)** dataset. We compared CpGs covered by at least 5 reads in both CCS and nanopore datasets. For coverage 5×-25×, we subsampled corresponding coverage reads from the total reads, and repeated the subsampling 5 times. Values in the table for coverage 5×-25× are the average and standard deviation of 5 repeated tests in "average±std" format. *r*: Pearson correlation; r^2: the coefficient of determination; *ρ*: Spearman correlation; RMSE: root mean square error.

coverage	method		*r*	r^2	*ρ*	RMSE
	read-level calling	site-level calling				
5×	primrose	pb-CpG-tools (count)	0.798±0.0003	0.6369±0.0005	0.7378±0.0004	0.2037±0.0001
	ccsmeth	ccsmeth (count)	0.8388±0.0003	0.7037±0.0005	0.7782±0.0003	0.187±0.0001
	ccsmeth	ccsmeth (count, Δ_p=0.33)	0.8505±0.0003	0.7234±0.0005	0.7875±0.0003	0.1875±0.0001
	primrose	pb-CpG-tools (model)	0.8248±0.0003	0.6803±0.0005	0.7662±0.0003	0.2228±0.0001
	primrose	ccsmeth (model)	0.8626±0.0003	0.7441±0.0005	0.8135±0.0002	0.1683±0.0001
	ccsmeth	ccsmeth (model)	0.8881±0.0002	0.7887±0.0004	0.8411±0.0002	0.1554±0.0001
10×	primrose	pb-CpG-tools (count)	0.8331±0.0001	0.6941±0.0001	0.7705±0.0001	0.1845±0
	ccsmeth	ccsmeth (count)	0.8685±0.0001	0.7543±0.0001	0.8065±0.0001	0.1674±0
	ccsmeth	ccsmeth (count, Δ_p=0.33)	0.8795±0.0001	0.7735±0.0001	0.8139±0.0001	0.1655±0
	primrose	pb-CpG-tools (model)	0.8629±0.0001	0.7446±0.0001	0.7994±0.0001	0.1952±0
	primrose	ccsmeth (model)	0.8835±0.0001	0.7806±0.0002	0.8332±0.0001	0.1561±0
	ccsmeth	ccsmeth (model)	0.9062±0.0001	0.8212±0.0001	0.8583±0.0001	0.1433±0
15×	primrose	pb-CpG-tools (count)	0.8557±0	0.7323±0.0001	0.7928±0.0001	0.1714±0
	ccsmeth	ccsmeth (count)	0.887±0	0.7867±0.0001	0.8262±0	0.1541±0
	ccsmeth	ccsmeth (count, Δ_p=0.33)	0.8971±0	0.8049±0.0001	0.833±0	0.1508±0
	primrose	pb-CpG-tools (model)	0.8847±0	0.7828±0.0001	0.8185±0.0001	0.1761±0
	primrose	ccsmeth (model)	0.8959±0	0.8026±0.0001	0.8469±0.0001	0.1477±0
	ccsmeth	ccsmeth (model)	0.9168±0	0.8405±0.0001	0.8704±0.0001	0.1351±0
20×	primrose	pb-CpG-tools (count)	0.8698±0	0.7565±0.0001	0.8075±0.0001	0.1634±0
	ccsmeth	ccsmeth (count)	0.8984±0	0.807±0.0001	0.8393±0.0001	0.1459±0
	ccsmeth	ccsmeth (count, Δ_p=0.33)	0.9077±0	0.824±0.0001	0.8458±0.0001	0.1417±0
	primrose	pb-CpG-tools (model)	0.897±0	0.8047±0.0001	0.83±0.0001	0.1641±0
	primrose	ccsmeth (model)	0.9037±0	0.8166±0.0001	0.856±0.0001	0.1422±0
	ccsmeth	ccsmeth (model)	0.9233±0	0.8525±0.0001	0.8783±0.0001	0.1298±0
25×	primrose	pb-CpG-tools (count)	0.8794±0	0.7733±0.0001	0.8178±0	0.1581±0
	ccsmeth	ccsmeth (count)	0.906±0	0.8209±0	0.8485±0	0.1403±0
	ccsmeth	ccsmeth (count, Δ_p=0.33)	0.9149±0	0.837±0	0.855±0	0.1356±0
	primrose	pb-CpG-tools (model)	0.9049±0	0.8189±0.0001	0.8395±0	0.1561±0
	primrose	ccsmeth (model)	0.9091±0	0.8264±0.0001	0.8624±0	0.1382±0
	ccsmeth	ccsmeth (model)	0.9278±0	0.8608±0	0.8839±0	0.126±0
25.6×(all)	primrose	pb-CpG-tools (count)	0.8813	0.7768	0.8200	0.1569
	ccsmeth	ccsmeth (count)	0.9076	0.8238	0.8504	0.1391
	ccsmeth	ccsmeth (count, Δ_p=0.33)	0.9163	0.8396	0.8569	0.1343
	primrose	pb-CpG-tools (model)	0.9065	0.8218	0.8417	0.1545
	primrose	ccsmeth (model)	0.9102	0.8284	0.8637	0.1373
	ccsmeth	ccsmeth (model)	0.9287	0.8626	0.8850	0.1252

Supplementary Table 7 Evaluation of ccsmeth and primrose at genome-wide site level against **BS-seq** on **HG002 (20Kb)** dataset. We compared CpGs covered by at least 5 reads in both CCS and BS-seq datasets. For coverage 5×-15×, we subsampled corresponding coverage reads from the total reads, and repeated the subsampling 5 times. Values in the table for coverage 5×-15× are the average and standard deviation of 5 repeated tests in "average±std" format. r: Pearson correlation; r^2: the coefficient of determination; ρ: Spearman correlation; RMSE: root mean square error.

coverage	method		r	r^2	ρ	RMSE
	read-level calling	site-level calling				
5×	primrose	pb-CpG-tools (count)	0.78±0.0002	0.6084±0.0004	0.706±0.0004	0.2205±0
	ccsmeth	ccsmeth (count)	0.8253±0.0003	0.6811±0.0005	0.7449±0.0005	0.2002±0.0001
	ccsmeth	ccsmeth (count, Δ_p=0.33)	0.8378±0.0003	0.7018±0.0005	0.7548±0.0005	0.1985±0.0001
	primrose	pb-CpG-tools (model)	0.8165±0.0002	0.6667±0.0003	0.7425±0.0003	0.2266±0.0001
	primrose	ccsmeth (model)	0.8406±0.0003	0.7067±0.0006	0.7806±0.0005	0.1906±0.0001
	ccsmeth	ccsmeth (model)	0.8842±0.0003	0.7817±0.0005	0.8283±0.0004	0.1636±0.0001
10×	primrose	pb-CpG-tools (count)	0.8247±0.0001	0.6802±0.0002	0.7462±0.0002	0.2008±0
	ccsmeth	ccsmeth (count)	0.8606±0.0001	0.7407±0.0001	0.7763±0.0001	0.1805±0
	ccsmeth	ccsmeth (count, Δ_p=0.33)	0.8733±0.0001	0.7626±0.0001	0.7852±0.0001	0.1748±0
	primrose	pb-CpG-tools (model)	0.8653±0.0001	0.7487±0.0001	0.7864±0.0001	0.1931±0.0001
	primrose	ccsmeth (model)	0.8726±0.0001	0.7614±0.0002	0.8071±0.0002	0.1748±0.0001
	ccsmeth	ccsmeth (model)	0.9083±0.0001	0.8251±0.0001	0.8499±0.0001	0.1482±0.0001
15×	primrose	pb-CpG-tools (count)	0.8549±0	0.7308±0.0001	0.775±0.0001	0.1866±0
	ccsmeth	ccsmeth (count)	0.8829±0	0.7794±0	0.7989±0.0001	0.1667±0
	ccsmeth	ccsmeth (count, Δ_p=0.33)	0.895±0	0.8011±0	0.8075±0.0001	0.1584±0
	primrose	pb-CpG-tools (model)	0.8947±0	0.8004±0	0.8163±0.0001	0.1686±0
	primrose	ccsmeth (model)	0.8924±0.0001	0.7964±0.0001	0.8263±0.0001	0.1629±0.0001
	ccsmeth	ccsmeth (model)	0.9224±0	0.8509±0	0.8653±0.0001	0.1372±0
17.0×(all)	primrose	pb-CpG-tools (count)	0.8648	0.7479	0.7849	0.1819
	ccsmeth	ccsmeth (count)	0.8900	0.7922	0.8066	0.1622
	ccsmeth	ccsmeth (count, Δ_p=0.33)	0.9019	0.8134	0.8152	0.1531
	primrose	pb-CpG-tools (model)	0.9038	0.8168	0.8265	0.1603
	primrose	ccsmeth (model)	0.8991	0.8084	0.8334	0.1586
	ccsmeth	ccsmeth (model)	0.9271	0.8594	0.8707	0.1333

Supplementary Table 8 Evaluation of ccsmeth and primrose at genome-wide site level against **nanopore sequencing** on **HG002 (20Kb)** dataset. We compared CpGs covered by at least 5 reads in both CCS and nanopore datasets. For coverage 5×-15×, we subsampled corresponding coverage reads from the total reads, and repeated the subsampling 5 times. Values in the table for coverage 5×-15× are the average and standard deviation of 5 repeated tests in "average±std" format. r: Pearson correlation; r^2: the coefficient of determination; ρ: Spearman correlation; RMSE: root mean square error.

coverage	method		r	r^2	ρ	RMSE
	read-level calling	site-level calling				
5×	primrose	pb-CpG-tools (count)	0.7647±0.0003	0.5847±0.0004	0.7019±0.0004	0.2159±0
	ccsmeth	ccsmeth (count)	0.8192±0.0003	0.6711±0.0004	0.7554±0.0004	0.195±0.0001
	ccsmeth	ccsmeth (count, Δ_p=0.33)	0.8306±0.0003	0.6898±0.0004	0.7636±0.0004	0.1981±0.0001
	primrose	pb-CpG-tools (model)	0.7967±0.0003	0.6348±0.0004	0.733±0.0004	0.2376±0.0001
	primrose	ccsmeth (model)	0.8262±0.0004	0.6827±0.0006	0.7691±0.0006	0.1861±0.0001
	ccsmeth	ccsmeth (model)	0.8749±0.0003	0.7655±0.0005	0.8242±0.0003	0.1608±0.0001
10×	primrose	pb-CpG-tools (count)	0.8061±0.0001	0.6498±0.0002	0.7389±0.0001	0.1968±0.0001
	ccsmeth	ccsmeth (count)	0.8522±0.0001	0.7263±0.0001	0.7851±0.0001	0.1759±0
	ccsmeth	ccsmeth (count, Δ_p=0.33)	0.8635±0.0001	0.7457±0.0002	0.7916±0.0001	0.1757±0.0001
	primrose	pb-CpG-tools (model)	0.8422±0.0001	0.7092±0.0002	0.77±0.0001	0.2078±0.0001
	primrose	ccsmeth (model)	0.8549±0.0002	0.7308±0.0003	0.792±0.0002	0.1725±0.0001
	ccsmeth	ccsmeth (model)	0.8967±0.0001	0.804±0.0002	0.8424±0.0001	0.1481±0.0001
15×	primrose	pb-CpG-tools (count)	0.8327±0.0001	0.6934±0.0001	0.7645±0.0001	0.1834±0
	ccsmeth	ccsmeth (count)	0.8723±0.0001	0.7608±0.0001	0.8061±0.0001	0.1628±0
	ccsmeth	ccsmeth (count, Δ_p=0.33)	0.883±0.0001	0.7798±0.0001	0.8122±0.0001	0.1608±0
	primrose	pb-CpG-tools (model)	0.8679±0.0001	0.7532±0.0001	0.7929±0.0001	0.1873±0
	primrose	ccsmeth (model)	0.8718±0.0001	0.76±0.0001	0.8087±0.0001	0.1627±0
	ccsmeth	ccsmeth (model)	0.9087±0	0.8258±0.0001	0.8555±0.0001	0.1395±0
17.0×(all)	primrose	pb-CpG-tools (count)	0.8416	0.7084	0.7735	0.1789
	ccsmeth	ccsmeth (count)	0.8788	0.7724	0.8135	0.1584
	ccsmeth	ccsmeth (count, Δ_p=0.33)	0.8893	0.7909	0.8196	0.1559
	primrose	pb-CpG-tools (model)	0.8758	0.7671	0.8008	0.1804
	primrose	ccsmeth (model)	0.8776	0.7701	0.8152	0.1591
	ccsmeth	ccsmeth (model)	0.9127	0.8330	0.8602	0.1365

Supplementary Table 9 Evaluation of ccsmeth and primrose at genome-wide site level against **BS-seq** on **HG002 (24Kb)** dataset. We compared CpGs covered by at least 5 reads in both CCS and BS-seq datasets. For coverage 5×-25×, we subsampled corresponding coverage reads from the total reads, and repeated the subsampling 5 times. Values in the table for coverage 5×-25× are the average and standard deviation of 5 repeated tests in "average±std" format. r: Pearson correlation; r^2: the coefficient of determination; ρ: Spearman correlation; RMSE: root mean square error.

coverage	method		r	r^2	ρ	RMSE
	read-level calling	site-level calling				
5×	primrose	pb-CpG-tools (count)	0.7818±0.0001	0.6112±0.0002	0.7156±0.0003	0.2217±0
	ccsmeth	ccsmeth (count)	0.8249±0.0001	0.6805±0.0002	0.7524±0.0003	0.2026±0.0001
	ccsmeth	ccsmeth (count, Δ_p=0.33)	0.8395±0.0001	0.7047±0.0002	0.7631±0.0004	0.1985±0.0001
	primrose	pb-CpG-tools (model)	0.8247±0.0001	0.6801±0.0002	0.7571±0.0003	0.2221±0.0001
	primrose	ccsmeth (model)	0.8586±0.0002	0.7372±0.0003	0.8097±0.0003	0.181±0.0001
	ccsmeth	ccsmeth (model)	0.8864±0.0002	0.7857±0.0003	0.836±0.0003	0.1645±0.0001
10×	primrose	pb-CpG-tools (count)	0.8246±0	0.6799±0.0001	0.757±0.0001	0.2017±0
	ccsmeth	ccsmeth (count)	0.8605±0.0001	0.7404±0.0001	0.7873±0.0001	0.1822±0
	ccsmeth	ccsmeth (count, Δ_p=0.33)	0.8753±0.0001	0.7661±0.0002	0.797±0.0001	0.1734±0
	primrose	pb-CpG-tools (model)	0.8696±0.0001	0.7562±0.0001	0.8006±0.0001	0.1898±0
	primrose	ccsmeth (model)	0.8846±0.0001	0.7826±0.0002	0.8363±0.0002	0.166±0.0001
	ccsmeth	ccsmeth (model)	0.9087±0.0001	0.8257±0.0002	0.8591±0.0002	0.1494±0.0001
15×	primrose	pb-CpG-tools (count)	0.8545±0	0.7301±0.0001	0.7862±0	0.1877±0
	ccsmeth	ccsmeth (count)	0.8841±0.0001	0.7817±0.0001	0.8118±0	0.1682±0
	ccsmeth	ccsmeth (count, Δ_p=0.33)	0.8984±0.0001	0.8072±0.0001	0.8213±0.0001	0.1562±0
	primrose	pb-CpG-tools (model)	0.8983±0.0001	0.807±0.0001	0.8298±0.0001	0.1652±0
	primrose	ccsmeth (model)	0.9017±0.0001	0.8131±0.0001	0.8546±0.0001	0.1546±0.0001
	ccsmeth	ccsmeth (model)	0.923±0.0001	0.8519±0.0002	0.8749±0.0001	0.1381±0.0001
20×	primrose	pb-CpG-tools (count)	0.8729±0	0.762±0.0001	0.8047±0	0.1793±0
	ccsmeth	ccsmeth (count)	0.8985±0	0.8073±0.0001	0.8273±0.0001	0.1597±0
	ccsmeth	ccsmeth (count, Δ_p=0.33)	0.9121±0	0.8319±0.0001	0.8369±0.0001	0.1457±0
	primrose	pb-CpG-tools (model)	0.9148±0	0.8368±0.0001	0.8479±0.0001	0.1496±0
	primrose	ccsmeth (model)	0.9124±0.0001	0.8324±0.0001	0.8664±0.0001	0.1468±0
	ccsmeth	ccsmeth (model)	0.9318±0	0.8682±0	0.885±0	0.1305±0
25×	primrose	pb-CpG-tools (count)	0.8851±0	0.7834±0	0.817±0	0.1738±0
	ccsmeth	ccsmeth (count)	0.9078±0	0.8241±0.0001	0.8377±0	0.1541±0
	ccsmeth	ccsmeth (count, Δ_p=0.33)	0.9208±0	0.8478±0.0001	0.8474±0	0.1389±0
	primrose	pb-CpG-tools (model)	0.925±0	0.8556±0	0.8608±0	0.1394±0
	primrose	ccsmeth (model)	0.9194±0.0001	0.8453±0.0001	0.8745±0.0001	0.1413±0
	ccsmeth	ccsmeth (model)	0.9376±0	0.879±0.0001	0.8918±0.0001	0.1252±0
28.4×(all)	primrose	pb-CpG-tools (count)	0.8923	0.7962	0.8244	0.1705
	ccsmeth	ccsmeth (count)	0.9132	0.8340	0.8439	0.1508
	ccsmeth	ccsmeth (count, Δ_p=0.33)	0.9259	0.8572	0.8538	0.1348
	primrose	pb-CpG-tools (model)	0.9309	0.8666	0.8692	0.1333
	primrose	ccsmeth (model)	0.9237	0.8532	0.8794	0.1378
	ccsmeth	ccsmeth (model)	0.9410	0.8855	0.8960	0.1220

Supplementary Table 10 Evaluation of ccsmeth and primrose at genome-wide site level against **nanopore sequencing** on **HG002 (24Kb)** dataset. We compared CpGs covered by at least 5 reads in both CCS and nanopore datasets. For coverage 5×-25×, we subsampled corresponding coverage reads from the total reads, and repeated the subsampling 5 times. Values in the table for coverage 5×-25× are the average and standard deviation of 5 repeated tests in "average±std" format. *r*: Pearson correlation; r^2: the coefficient of determination; *ρ*: Spearman correlation; RMSE: root mean square error.

coverage	method		*r*	r^2	*ρ*	RMSE
	read-level calling	site-level calling				
5×	primrose	pb-CpG-tools (count)	0.766±0.0002	0.5868±0.0003	0.709±0.0004	0.2163±0.0001
	ccsmeth	ccsmeth (count)	0.8165±0.0002	0.6667±0.0003	0.7575±0.0003	0.1964±0.0001
	ccsmeth	ccsmeth (count, Δ_p=0.33)	0.8299±0.0002	0.6888±0.0004	0.7668±0.0003	0.1975±0.0001
	primrose	pb-CpG-tools (model)	0.8042±0.0002	0.6467±0.0003	0.7456±0.0003	0.2341±0.0001
	primrose	ccsmeth (model)	0.8432±0.0002	0.711±0.0003	0.7932±0.0003	0.1782±0.0001
	ccsmeth	ccsmeth (model)	0.8752±0.0002	0.7659±0.0004	0.8267±0.0002	0.1616±0.0001
10×	primrose	pb-CpG-tools (count)	0.8058±0.0001	0.6493±0.0001	0.7472±0.0001	0.1969±0
	ccsmeth	ccsmeth (count)	0.8501±0.0001	0.7226±0.0002	0.7904±0.0001	0.1765±0.0001
	ccsmeth	ccsmeth (count, Δ_p=0.33)	0.8635±0.0001	0.7457±0.0002	0.7981±0.0001	0.1738±0.0001
	primrose	pb-CpG-tools (model)	0.8462±0.0001	0.7161±0.0002	0.7823±0.0001	0.2054±0
	primrose	ccsmeth (model)	0.8663±0.0001	0.7504±0.0001	0.8157±0.0001	0.1659±0
	ccsmeth	ccsmeth (model)	0.8952±0.0001	0.8014±0.0001	0.8461±0.0001	0.1491±0
15×	primrose	pb-CpG-tools (count)	0.8324±0.0001	0.6928±0.0002	0.7731±0.0001	0.1836±0.0001
	ccsmeth	ccsmeth (count)	0.8717±0.0001	0.7598±0.0002	0.8132±0.0001	0.1628±0.0001
	ccsmeth	ccsmeth (count, Δ_p=0.33)	0.8846±0.0001	0.7824±0.0002	0.8205±0.0001	0.1578±0.0001
	primrose	pb-CpG-tools (model)	0.8715±0.0001	0.7595±0.0002	0.8043±0.0001	0.1849±0.0001
	primrose	ccsmeth (model)	0.8807±0.0001	0.7756±0.0002	0.8313±0.0001	0.1571±0.0001
	ccsmeth	ccsmeth (model)	0.9076±0.0001	0.8237±0.0001	0.8596±0.0001	0.1403±0.0001
20×	primrose	pb-CpG-tools (count)	0.8492±0	0.7211±0.0001	0.79±0	0.1754±0
	ccsmeth	ccsmeth (count)	0.8852±0	0.7836±0	0.828±0	0.1543±0
	ccsmeth	ccsmeth (count, Δ_p=0.33)	0.8973±0	0.8052±0	0.8353±0	0.1478±0
	primrose	pb-CpG-tools (model)	0.8862±0	0.7853±0	0.8183±0	0.1718±0
	primrose	ccsmeth (model)	0.89±0	0.792±0	0.8417±0	0.1511±0
	ccsmeth	ccsmeth (model)	0.9154±0	0.838±0	0.8684±0	0.1343±0
25×	primrose	pb-CpG-tools (count)	0.8605±0.0001	0.7405±0.0001	0.8015±0.0001	0.17±0
	ccsmeth	ccsmeth (count)	0.8942±0	0.7996±0.0001	0.8382±0.0001	0.1486±0
	ccsmeth	ccsmeth (count, Δ_p=0.33)	0.9057±0	0.8204±0.0001	0.8456±0.0001	0.1412±0
	primrose	pb-CpG-tools (model)	0.8955±0.0001	0.802±0.0001	0.8293±0.0001	0.1631±0
	primrose	ccsmeth (model)	0.8963±0	0.8033±0.0001	0.8488±0.0001	0.1469±0
	ccsmeth	ccsmeth (model)	0.9207±0	0.8477±0.0001	0.8744±0.0001	0.1301±0
28.4×(all)	primrose	pb-CpG-tools (count)	0.8674	0.7524	0.8086	0.1668
	ccsmeth	ccsmeth (count)	0.8996	0.8093	0.8445	0.1452
	ccsmeth	ccsmeth (count, Δ_p=0.33)	0.9108	0.8295	0.8520	0.1372
	primrose	pb-CpG-tools (model)	0.9011	0.8119	0.8370	0.1579
	primrose	ccsmeth (model)	0.9002	0.8103	0.8532	0.1442
	ccsmeth	ccsmeth (model)	0.9240	0.8537	0.8781	0.1275

Supplementary Table 11 Evaluation of ccsmeth and primrose at genome-wide site level against **BS-seq** on **SD0651_P1 (15Kb)** dataset. We compared CpGs covered by at least 5 reads in both CCS and BS-seq datasets. For coverage 5×-15×, we subsampled corresponding coverage reads from the total reads, and repeated the subsampling 5 times. Values in the table for coverage 5×-15× are the average and standard deviation of 5 repeated tests in "average±std" format. r: Pearson correlation; r^2: the coefficient of determination; ρ: Spearman correlation; RMSE: root mean square error.

coverage	method		r	r^2	ρ	RMSE
	read-level calling	site-level calling				
5×	primrose	pb-CpG-tools (count)	0.667±0.0007	0.4449±0.0009	0.3993±0.0005	0.2279±0
	ccsmeth	ccsmeth (count)	0.716±0.0007	0.5127±0.0009	0.4463±0.0006	0.2095±0
	ccsmeth	ccsmeth (count, Δ_p=0.33)	0.7367±0.0006	0.5427±0.0009	0.4581±0.0006	0.1992±0
	primrose	pb-CpG-tools (model)	0.7738±0.0006	0.5988±0.001	0.4598±0.0007	0.1772±0.0001
	primrose	ccsmeth (model)	0.7528±0.0006	0.5667±0.001	0.4372±0.0006	0.1897±0
	ccsmeth	ccsmeth (model)	0.8233±0.0005	0.6778±0.0008	0.5012±0.0006	0.1554±0
10×	primrose	pb-CpG-tools (count)	0.7218±0.0002	0.521±0.0003	0.4305±0.0001	0.2095±0
	ccsmeth	ccsmeth (count)	0.7633±0.0002	0.5826±0.0003	0.4742±0.0002	0.1913±0
	ccsmeth	ccsmeth (count, Δ_p=0.33)	0.7849±0.0002	0.6161±0.0003	0.4831±0.0002	0.1775±0
	primrose	pb-CpG-tools (model)	0.8213±0.0002	0.6745±0.0003	0.4807±0.0002	0.1568±0
	primrose	ccsmeth (model)	0.7873±0.0002	0.6198±0.0004	0.4551±0.0001	0.1781±0.0001
	ccsmeth	ccsmeth (model)	0.8506±0.0002	0.7235±0.0004	0.521±0.0002	0.1437±0.0001
15×	primrose	pb-CpG-tools (count)	0.7598±0.0001	0.5773±0.0001	0.4542±0.0001	0.1969±0
	ccsmeth	ccsmeth (count)	0.794±0.0001	0.6304±0.0001	0.4964±0	0.1792±0
	ccsmeth	ccsmeth (count, Δ_p=0.33)	0.815±0.0001	0.6643±0.0001	0.5042±0.0001	0.1632±0
	primrose	pb-CpG-tools (model)	0.8501±0	0.7226±0.0001	0.493±0.0001	0.1416±0
	primrose	ccsmeth (model)	0.809±0.0001	0.6544±0.0002	0.4688±0.0002	0.1691±0.0001
	ccsmeth	ccsmeth (model)	0.8653±0.0001	0.7488±0.0001	0.5358±0	0.1359±0
19.6×(all)	primrose	pb-CpG-tools (count)	0.7839	0.6146	0.4707	0.1893
	ccsmeth	ccsmeth (count)	0.8128	0.6607	0.5117	0.1720
	ccsmeth	ccsmeth (count, Δ_p=0.33)	0.8329	0.6937	0.5197	0.1548
	primrose	pb-CpG-tools (model)	0.8702	0.7572	0.5093	0.1274
	primrose	ccsmeth (model)	0.8239	0.6788	0.4800	0.1624
	ccsmeth	ccsmeth (model)	0.8750	0.7656	0.5461	0.1303

Supplementary Table 12 Evaluation of ccsmeth and primrose at genome-wide site level against **nanopore sequencing** on **CHM13 (20Kb)** dataset. We compared CpGs covered by at least 5 reads in both CCS and nanopore datasets. For coverage 5×-15×, we subsampled corresponding coverage reads from the total reads, and repeated the subsampling 5 times. Values in the table for coverage 5×-15× are the average and standard deviation of 5 repeated tests in "average±std" format. *r*: Pearson correlation; r^2: the coefficient of determination; *ρ*: Spearman correlation; RMSE: root mean square error.

coverage	method		*r*	r^2	*ρ*	RMSE
	read-level calling	site-level calling				
5×	primrose	pb-CpG-tools (count)	0.7941±0.0001	0.6305±0.0002	0.7621±0.0004	0.2192±0
	ccsmeth	ccsmeth (count)	0.8359±0.0002	0.6987±0.0003	0.8124±0.0003	0.2±0.0001
	ccsmeth	ccsmeth (count, Δ_p=0.33)	0.849±0.0002	0.7208±0.0003	0.8206±0.0003	0.2013±0.0001
	primrose	pb-CpG-tools (model)	0.8332±0.0002	0.6943±0.0003	0.8029±0.0003	0.2334±0.0001
	primrose	ccsmeth (model)	0.8698±0.0001	0.7566±0.0002	0.8248±0.0003	0.1759±0.0001
	ccsmeth	ccsmeth (model)	0.8989±0.0001	0.808±0.0002	0.8643±0.0002	0.1587±0.0001
10×	primrose	pb-CpG-tools (count)	0.8357±0.0001	0.6984±0.0001	0.8032±0.0001	0.1967±0
	ccsmeth	ccsmeth (count)	0.8692±0.0001	0.7556±0.0002	0.8456±0.0002	0.1779±0.0001
	ccsmeth	ccsmeth (count, Δ_p=0.33)	0.8816±0.0001	0.7772±0.0001	0.8521±0.0001	0.1756±0
	primrose	pb-CpG-tools (model)	0.8787±0.0001	0.7721±0.0001	0.8427±0.0001	0.1966±0
	primrose	ccsmeth (model)	0.8939±0.0001	0.7991±0.0001	0.8479±0.0001	0.1608±0
	ccsmeth	ccsmeth (model)	0.9181±0.9181	0.843±0.843	0.8848±0.8848	0.1448±0.1448
15×	primrose	pb-CpG-tools (count)	0.8639±0	0.7462±0	0.8286±0	0.181±0
	ccsmeth	ccsmeth (count)	0.8911±0	0.794±0	0.8667±0	0.1622±0
	ccsmeth	ccsmeth (count, Δ_p=0.33)	0.9024±0	0.8143±0.0001	0.8724±0.0001	0.1575±0
	primrose	pb-CpG-tools (model)	0.905±0	0.8191±0.0001	0.864±0	0.1714±0
	primrose	ccsmeth (model)	0.9087±0	0.8257±0	0.8605±0	0.1499±0
	ccsmeth	ccsmeth (model)	0.9299±0	0.8647±0	0.8962±0	0.135±0
16.5×(all)	primrose	pb-CpG-tools (count)	0.8711	0.7589	0.8350	0.1770
	ccsmeth	ccsmeth (count)	0.8966	0.8039	0.8720	0.1582
	ccsmeth	ccsmeth (count, Δ_p=0.33)	0.9075	0.8236	0.8775	0.1529
	primrose	pb-CpG-tools (model)	0.9112	0.8303	0.8688	0.1650
	primrose	ccsmeth (model)	0.9124	0.8325	0.8636	0.1469
	ccsmeth	ccsmeth (model)	0.9328	0.8701	0.8990	0.1324

Supplementary Table 13 Comparing CCS with BS-seq and nanopore sequencing on predicting site-level methylation frequencies of HG002 haplotypes phased by Illumina trio data. CpGs in haplotypes of autosomes were used for evaluation. *r*: Pearson correlation; r^2: the coefficient of determination; *ρ*: Spearman correlation; RMSE: root mean square error; ONT: nanopore sequencing.

benchmark	haplotype	*r*	r^2	*ρ*	RMSE
BS-seq	maternal	0.9321	0.8689	0.8505	0.1366
	paternal	0.9322	0.8689	0.8507	0.1365
ONT	maternal	0.9403	0.8842	0.8623	0.1215
	paternal	0.9404	0.8844	0.8625	0.1214

Supplementary Table 14 The number of CpGs in autosomes and sex chromosomes detected by using difference coverage of HG002 CCS reads. Values for 5×-70× are the average and standard deviation of 5 repeated tests in "average±std" format.

sample	data_type	coverage	number of CpGs
HG002	CCS	5	16355322.6±32213.69
HG002	CCS	10	29168678.8±13633.79
HG002	CCS	15	31331716±6140.61
HG002	CCS	20	31907365.2±2354.58
HG002	CCS	25	32152455.2±5660.62
HG002	CCS	30	32295541.2±4856.94
HG002	CCS	35	32392385±4284.79
HG002	CCS	40	32464914.6±3946.71
HG002	CCS	45	32525561±2717.7
HG002	CCS	50	32577563.6±1736.5
HG002	CCS	55	32621611.4±1994.63
HG002	CCS	60	32658534.6±2874.86
HG002	CCS	65	32691926.6±1646.07
HG002	CCS	70	32721946.4±1213.63
HG002	CCS	71.0	32737721

Supplementary Table 15 The number of CpGs in autosomes phased by using difference coverage of HG002 CCS reads. Values for 5×-70× are the average and standard deviation of 5 repeated tests in "average±std" format.

sample	data_type	coverage	number of CpGs
HG002	CCS	5	16355322.6±32213.69
HG002	CCS	10	29168678.8±13633.79
HG002	CCS	15	31331716±6140.61
HG002	CCS	20	31907365.2±2354.58
HG002	CCS	25	32152455.2±5660.62
HG002	CCS	30	32295541.2±4856.94
HG002	CCS	35	32392385±4284.79
HG002	CCS	40	32464914.6±3946.71
HG002	CCS	45	32525561±2717.7
HG002	CCS	50	32577563.6±1736.5
HG002	CCS	55	32621611.4±1994.63
HG002	CCS	60	32658534.6±2874.86
HG002	CCS	65	32691926.6±1646.07
HG002	CCS	70	32721946.4±1213.63
HG002	CCS	71.0	32737721

Supplementary Table 16 Comparing CCS (ccsmeth) with BS-seq (Bismark) and nanopore sequencing (DeepSignal2) on predicting site-level methylation frequencies in repetitive genomic regions using HG002 data. r: Pearson correlation; r^2: the coefficient of determination; ρ: Spearman correlation; RMSE: root mean square error; ONT: nanopore sequencing; RepeatMasker: repetitive genomic elements annotated by RepeatMasker; SDs: segmental duplications; cenSats: peri/centromeric satellites.

region	benchmark	r	r^2	ρ	RMSE
RepeatMasker	BS-seq	0.9540	0.9101	0.9102	0.1055
	ONT	0.9358	0.8758	0.8902	0.1138
SDs	BS-seq	0.9208	0.8479	0.8770	0.1370
	ONT	0.9087	0.8257	0.8791	0.1308
cenSats	BS-seq	0.8822	0.7783	0.8462	0.1584
	ONT	0.8572	0.7349	0.8327	0.1606

Supplementary Table 17 Comparing CCS (ccsmeth) with BS-seq (Bismark) and nanopore sequencing (DeepSignal2) on predicting site-level methylation frequencies in repetitive genomic regions of HG002 haplotypes phased by Illumina trio data. r: Pearson correlation; r^2: the coefficient of determination; ρ: Spearman correlation; RMSE: root mean square error; ONT: nanopore sequencing; RepeatMasker: repetitive genomic elements annotated by RepeatMasker; SDs: segmental duplications; cenSats: peri/centromeric satellites.

region	benchmark	haplotype	r	r^2	ρ	RMSE
RepeatMasker	BS-seq	maternal	0.9283	0.8617	0.8429	0.1370
		paternal	0.9285	0.8621	0.8432	0.1369
	ONT	maternal	0.9365	0.8771	0.8546	0.1221
		paternal	0.9368	0.8775	0.8550	0.1218
SDs	BS-seq	maternal	0.9053	0.8196	0.8332	0.1558
		paternal	0.9007	0.8113	0.8311	0.1599
	ONT	maternal	0.9175	0.8419	0.8490	0.1377
		paternal	0.9160	0.8390	0.8520	0.1389
cenSats	BS-seq	maternal	0.8907	0.7933	0.8340	0.1633
		paternal	0.8925	0.7966	0.8378	0.1628
	ONT	maternal	0.9023	0.8141	0.8553	0.1489
		paternal	0.9066	0.8219	0.8612	0.1458

Supplementary Table 18 Runtime (wall clock time) and peak memory usage for the main steps of HK model, primrose, and ccsmeth on processing **subsampled 100K ZMW reads** of HG002 15Kb, 20Kb, and 24Kb. Note that we only evaluate the steps for per-read methylation calling and the preprocessing steps since HK model does not support calculating site-level methylation frequency.

method	step	No. of CPU cores	No. of GPUs	runtime (h:mm:ss or m:ss)/peak memory (GB)		
				HG002 (15Kb)	HG002 (20Kb)	HG002 (24Kb)
HK model	Align	40	-	14:08/58.94	9:14/54.69	13:52/60.77
	Extract features	1	-	18:12:43/6.61	13:10:45/6.33	19:16:50/6.68
	Format matrix	1	-	24:09/18.99	23:24/16.64	29:34/23.56
	Predict	1	1	2:36/21.20	1:31/18.80	2:07/25.95
primrose	Call CCS (pbccs)	40	-	22:41/4.46	18:33/4.81	21:10/5.41
	Align (pbmm2)	40	-	1:20/25.29	1:18/25.12	1:26/26.09
	Call modifications	40	-	0:08/0.42	0:07/0.46	0:10/0.54
ccsmeth	Call CCS (pbccs)	40	-	22:41/4.46	18:33/4.81	21:10/5.41
	Align (pbmm2)	40	-	1:20/25.29	1:18/25.12	1:26/26.09
	Call modifications	40	1	5:57/2.44	4:24/2.47	6:42/2.56

Supplementary Table 19 Runtime (wall clock time) and peak memory usage for the main steps of primrose and ccsmeth on processing **1 SMRT cell reads** of HG002 15Kb, 20Kb, and 24Kb (cell ID: m64008_201124_002822 of HG002 15Kb, m64011_190901_095311 of HG002 20Kb, and m64014_200920_132517 of HG002 24Kb).

method	step		No. of CPU cores	No. of GPUs	runtime (h:mm:ss or m:ss)/peak memory (GB)		
					HG002 (15Kb)	HG002 (20Kb)	HG002 (24Kb)
primrose/ pb-cpg-tools	Call CCS (pbccs)		40	-	40:39:40/6.01	27:29:36/4.96	46:27:22/6.31
	Align (pbmm2)		40	-	58:18/65.54	37:40/65.26	1:13:07/68.64
	Call per-read methylation		40	-	8:55/1.22	5:45/1.25	10:24/1.56
	Call methylation frequency	count-mode	40	-	1:15:58/21.23	1:12:52/20.90	1:18:55/21.33
		model-mode	40	-	52:55/15.64	50:47/14.66	57:36/15.83
ccsmeth	Call CCS (pbccs)		40	-	40:39:40/6.01	27:29:36/4.96	46:27:22/6.31
	Align (pbmm2)		40	-	58:18/65.54	37:40/65.26	1:13:07/68.64
	Call per-read methylation		40	1	4:53:40/30.44	3:11:12/30.39	5:48:11/30.41
	Call methylation frequency	count-mode	40	-	15:12/18.58	12:10/18.46	17:19/18.59
		model-mode	40	-	55:36/18.58	45:29/18.46	59:18/18.59

Supplementary Table 20 Computing resources used for evaluating the runtime of the processes in ccsmethphase.

process	server	No. of CPU cores	No. of GPU cards
SAMTOOLS_index_bam	Server-CPU	40	-
CCSMETH_call_mods	Server-GPU	40	2
PBMM2	Server-CPU	40	-
SAMTOOLS_merge_bam	Server-CPU	40	-
CLAIR3	Server-CPU	40	-
WHATSHAP_phase_haplotag	Server-CPU	10	-
CCSMETH_call_freq	Server-CPU	40	-
DSS	Server-CPU	40	-

Supplementary Notes

Supplementary Note 1 Evaluation of ccsmeth for 5mCpG detection at read level in different genomic contexts and regions

Different genomic regions may vary in sequence contexts and methylation levels[8]. To explore whether the performance of ccsmeth is correlated with any genomic features, we further examine ccsmeth for read-level 5mCpG detection in different genomic contexts and regions using the datasets of HG002 and SD0651_P1.

We consider the following genomic contexts and regions: (1) Singletons and non-singletons. A CpG is called singleton if there are no other CpGs in the up and down 10 bp regions. Otherwise, it is called non-singleton. (2) Genic and intergenic regions. We examine three genic regions, including promoters, exons, and introns. For exons and introns, we extract corresponding regions from the gene annotation file of CHM13 v2.0. We extend the regions of transcription start sites (TSS) 2000 bp up and 200 bp down as promoters. Then, we take regions which are not included in exons, introns, and promoters as intergenic regions. (3) CpG islands, shores, and shelves. We download the CpG islands annotations of CHM13 v2.0. We take the regions located 2000 bp up and down from CpG islands as CpG shores. Then we take regions located 2000 bp up and down from CpG shores as CpG shelves. (4) Repetitive regions. Based on the RepeatMasker annotations of CHM13 v2.0, we examined five categories of repetitive regions: Simple repeats, short interspersed nuclear elements (SINE), long interspersed nuclear element (LINE), long terminal repeat (LTR), and others (All repetitive regions other than simple repeats, SINE, LINE, LTR are taken as "others").

We use the high-confidence methylated and unmethylated CpGs of HG002 and SD0651_P1 for the read-level evaluation (Supplementary Fig. 3a). As shown in Supplementary Fig. 3b, compared to the genome-wide performance, ccsmeth has much higher accuracies in non-singletons and CpG islands but has lower accuracies in singletons, indicating that ccsmeth tends to have higher performances in regions with high CpG densities. ccsmeth has relative lower accuracies in intergenic regions, CpG shores, and CpG shelves. In simple repeats and "Others" repetitive regions, ccsmeth has lower sensitivities and specificities, respectively. On all four datasets, the results of primrose show consistent patterns with ccsmeth across all tested regions. The results indicate that biologically relevant genomic contexts and regions do impact the performance of 5mCpG detection. Further studies are needed to focus on improving the performance of 5mCpG detection in specific genomic regions.

Supplementary Note 2 Comparison of the count mode and model mode of ccsmeth

We use the HG002 CCS datasets (71.0×) to compare the methylation frequencies calculated by the count mode and model mode of ccsmeth:

Suppose R_b, R_c, R_m are the methylation frequencies of a CpG calculated by BS-seq, count mode of ccsmeth, and model mode of ccsmeth, respectively. We use $| R_b - R_c | - | R_b - R_m |$ to measure whether R_c or R_m is closer to R_b. If $| R_b - R_c | - | R_b - R_m | > 0.1$, meaning the model mode has a more accurate prediction than count mode, we classify the CpG into the group G_m. If $| R_b - R_c | - | R_b - R_m | < -0.1$, we classify the CpG into the group G_c. We find that among the total tested 29,174,320 CpGs, 3,975,014 CpGs are classified to G_m, while 644,370 CpGs are classified to G_c (Supplementary Fig. 7a). The methylation frequencies of CpGs in the two groups show significant differences: CpGs in G_m tend to have either very low (<0.2) or high (>0.8) methylation frequencies, while CpGs in G_c tend to have intermediate methylation frequencies (Supplementary Fig. 7b). The comparison of genome-wide per-site methylation frequency between the count mode and model mode of ccsmeth is shown in Supplementary Fig. 7c.

Supplementary Note 3 Pipeline for haplotype-aware methylation calling using Illumina whole-genome sequencing (WGS) trio data and BS-seq data

To evaluate the methylation phasing pipeline on CCS data, we performed haplotype-aware methylation calling using WGS and BS-seq reads of HG002 as the benchmark (Supplementary Fig. 8). In this pipeline, we used SNPsplit (version 0.5.0) [9] to assign BS-seq reads to the haplotypes of HG002. SNPsplit requires information of heterozygous SNVs of HG002 and the origin of these SNVs for accurate reads alignment and splitting. Thus, we downloaded the Illumina WGS reads of AshkenazimTrio: HG003 is the father, HG004 is the mother, HG002 is the son. We used BWA-MEM (version 0.7.17-r1194-dirty)[10] to align the WGS reads, and then used DeepTrio[11] (version 1.3.0) to call SNVs for HG002, HG003, and HG004. Then, we used the following rules as in NanoMethPhase[12] to phase heterozygous SNVs in autosomes of HG002:

$$\begin{cases} \text{Haplotype}(S) = 1, & if\ S \in \text{maternal SNVs}\ and\ S \notin \text{paternal SNVs}\ and\ S \in \text{child's heterozygous SNVs} \\ \text{Haplotype}(S) = 1, & else\ if\ S \in \text{maternal homozygous SNVs}\ and\ S \notin \text{paternal homozygous SNVs}\ and\ S \in \text{child's heterozygous SNVs} \\ \text{Haplotype}(S) = 2, & else\ if\ S \notin \text{maternal SNVs}\ and\ S \in \text{paternal SNVs}\ and\ S \in \text{child's heterozygous SNVs} \\ \text{Haplotype}(S) = 2, & else\ if\ S \notin \text{maternal homozygous SNVs}\ and\ S \in \text{paternal homozygous SNVs}\ and\ S \in \text{child's heterozygous SNVs} \end{cases} \quad (1)$$

where S represents an SNV. After phasing, we generated a chromosome-level SNV phasing result (*i.e.*, all heterozygous SNVs of HG002 inherited from HG004 (mother) were assigned to Haplotype 1, and all heterozygous SNVs inherited from HG003 (father) were assigned to Haplotype 2).

We used Bismark[13] to align BS-seq reads to genome reference. Then, we used SNPsplit to assign the aligned BS-seq reads to haplotypes. We got the methylation profile of each haplotype using Bismark. At last, we got differentially methylated regions (DMRs) of the two haplotypes using DSS (version 2.44.0)[14] (Supplementary Fig. 8).

Supplementary Note 4 Pipeline for haplotype-aware methylation calling using nanopore data

The pipeline for haplotype-aware methylation calling using nanopore data is similar to the pipeline using PacBio data (Supplementary Fig. 9). In this pipeline, we used Guppy (version 4.2.2+effbaf8) to basecall nanopore raw reads. We then used Tombo[15] (version 1.5.1) to re-squiggle the raw signals in nanopore reads to the reference genome, and then used DeepSignal2 (v0.1.2, https://github.com/PengNi/deepsignal2)[16] to call 5mCpGs. We used Clair3[17] (v0.1-r11 minor 2) with "*r941_prom_hac_g360+g422*" model to call variants. The called "PASS" SNVs were then used by WhatsHap[18] (version 1.4) to assign the reads to two haplotypes. After generating phased methylation profiles by DeepSignal2, we used DSS (version 2.44.0)[14] to get DMRs.

Supplementary Note 5 The model architecture of ccsmeth

(1) bidirectional GRU

A bidirectional GRU[19] layer includes a forward GRU and a backward GRU to catch both the forward and reverse flow of features. Suppose $x_1, x_2, \ldots, x_t$ are a sequence of features, each time step x_i contains four features: the nucleotide base, the mean IPD value, the mean PW value, and the number of subreads. A GRU cell will recursively calculate the hidden layer h as follows:

$$r_t = \text{sigmoid}(W_r[h_{t-1},\ x_t] + b_r) \quad (2)$$

$$z_t = \text{sigmoid}(W_z[h_{t-1},\ x_t] + b_z) \quad (3)$$

$$\hat{h}_t = \tanh(W_h \cdot [r_t h_{t-1}, x_t] + b_h) \quad (4)$$

$$h_t = (1 - z_t)h_{t-1} + z_t\hat{h}_t \quad (5)$$

where W and b are weight matrices and biases. x_t is the input feature; r_t is a reset gate; z_t is an update gate; h_t is the hidden state; and $\hat{h}_t$ represents information that needs to be updated in the current cell. The outputs of

forward and backward GRU are combined as:

$$h_{t,A} = h_{t,F} \oplus h_{t,B} \quad \textbf{(6)}$$

(2) Bahdanau attention

Bahdanau attention[20] receives all the hidden states of RNN cells and outputs context vector c_t as follows:

$$\text{score}(h_t, h_s) = \tanh(W_1 h_t + W_2 h_s) \quad \textbf{(7)}$$

$$a_{ts} = \text{softmax}(\text{score}(h_t, h_s)) \quad \textbf{(8)}$$

$$c_t = a_{ts} {h_t}^T \quad \textbf{(9)}$$

where h_t represents the hidden state in the output vector of BiGRU; h_s contains the final hidden state for an element in the sequence from GRU; W_1 and W_2 are weight matrices.

(3) Softmax activation function to output methylated/unmethylated probabilities

A softmax activation layer is used in ccsmeth to predict the methylated and unmethylated probabilities of one sample as follows:

$$\text{softmax}(x_i) = \frac{e^{x_i}}{\sum_{j=0}^{1} e^{x_j}}, i = 0 \; or \; 1 \quad \textbf{(10)}$$

where x_0 and x_1 are two outputs from the former fully connected layer, for calculating unmethylated and methylated probabilities, respectively.

(4) The cross-entropy loss function used for training the read-level model is as follows:

$$L_{CE} = z * -\log(y) + (1 - z) * -\log(1 - y) \quad \textbf{(11)}$$

where z is the true label vector and y is the predicted methylated probability vector from the softmax function.

(5) The mean squared error (MSE) loss function for training the site-level model is as follows:

$$L_{MSE} = \frac{\sum_{i=0}^{n} ({x_i}^2 - {y_i}^2)}{n} \quad \textbf{(12)}$$

where x_i is the predicted methylation frequency, y_i is the true methylation frequency, n is the number of samples.

Supplementary Note 6 Testing ccsmethphase using CCS data of the HN0641 family trio

We sequenced three human samples of a Chinese family trio using CCS, and got 2 SMRT cells of CCS reads for each of the three samples: HN0641_FA (father), HN0641_MO (mother), HN0641_S1 (son). We tested ccsmethphase using the CCS reads of the family trio. As shown in Supplementary Fig. 15a, the results indicate that the three samples have similar methylation levels. 11.9%, 12.0%, 10.4% CpGs of HN0641_FA, HN0641_MO, HN0641_S1, respectively, have low (≤0.3) methylation frequencies, while 75.4%, 73.4%, 80.6% CpGs of the three samples, respectively, have high (≥0.7) methylation frequencies.

We then examined the haplotype-aware methylation status of known imprinted regions in HN0641_S1. As shown in Supplementary Fig. 15b, the well-characterized imprinted intervals show large methylation differences between the two haplotypes (median=0.51). 14.0% of other known imprinted intervals also show large (>0.5) methylation differences. The result of HN0641_S1 is consistent with the results in HG002 and SD0651_P1.

Using ccsmethphase, we generated 2,813 DMRs from the CCS data of HN0641_S1. The DMRs cover 108 (52.9%) of the known imprinted intervals (*i.e.*, 108 known imprinted intervals are overlapped with the CCS-generated DMRs) (Supplementary Fig. 15c). Moreover, the haplotype phasing results of the family trio show that ccsmethphase not only can detect imprinted intervals, but also reveals the pattern of parental imprinting correctly. In the results of ccsmethphase, the maternally imprinted intervals (*e.g.*, *GNAS_Ex1A* and *PEG10*) in HN0641_S1 show high methylation levels in the haplotype inherited from mother, while the paternally imprinted intervals (e.g., *MEG3*) show high methylation levels in the haplotype inherited from father (Supplementary Figs. 16-18).

Supplementary Note 7 Computational efficiency of ccsmeth and the ccsmethphase pipeline

We compared the runtime (wall clock time) and peak memory of ccsmeth with HK model and primrose. The comparison was performed at an HPC cluster containing two kinds of servers: (1) Server-CPU with 48 CPU cores (Intel(R) Xeon(R) Gold 6248R CPU @ 3.0GHz) and 192 GB RAM; (2) Server-GPU with 40 CPU cores (Intel(R) Xeon(R) Gold 6248 CPU @ 2.50GHz), 384 GB RAM, and 2 Nvidia Tesla V100 GPU cards. As shown in Supplementary Fig. 22, ccsmeth and primrose (+pb_CpG_tools) contain the same steps for methylation calling, while HK model has a different pipeline. The differences are mainly in the following aspects: (1) HK model extracts features from subreads, while ccsmeth and primrose take CCS reads as input. (2) HK model needs aligned reads for feature extraction, while the "*Call per-read methylation*" step of ccsmeth and primrose can be performed before or after the "*Align*" step. (3) There is no module or script in HK model for calculating site-level methylation frequency.

We first subsampled 100K ZMW reads from each of the three HG002 CCS datasets (15Kb, 20Kb, 24Kb) to compare all three methods. As shown in Supplementary Table 18, HK model is very time-consuming, especially in the "*Extract features*" step. This is mainly because HK model directly extracts features from subreads, and the script of HK model for "*Extract features*" is not optimized for parallel processing. We further used 1 SMRT cell CCS reads from each of the three HG002 datasets to compare primrose and ccsmeth (Supplementary Table 19). As shown in Supplementary Tables 18-19, primrose is extremely fast in calling per-read methylation. primrose takes ~6-10 minutes to call per-read methylation from 1 SMRT cell of CCS data, while ccsmeth needs ~3-6 hours. However, when CCS reads have been called from the raw subreads, the whole pipeline of ccsmeth takes at most 8 hours to call methylation from 1 SMRT cell CCS data. Compared to primrose which takes at most ~2.4 hours, ccsmeth can also be used in practice. In the future, we will continue to optimize ccsmeth in terms of computational efficiency.

We also evaluated the runtime of 8 main processes in the ccsmethphase pipeline: *SAMTOOLS_index_bam* for indexing the CCS bam files, *CCSMETH_call_mods* for calling methylation in CCS reads, *PBMM2* for aligning CCS reads to the reference genome, *SAMTOOLS_merge_bam* for merging alignment bam files of the same "sample", *CLAIR3* for calling SNVs, *WHATSHAP_phase_haplotag* for phasing SNVs and reads, *CCSMETH_call_freq* for calling methylation frequencies of CpGs, and *DSS* for calling DMRs. The data used for evaluation include 10 SMRT cells of CCS reads used for testing in this study, in which there are 2 SMRT cells of CCS reads for each of the 5 "samples": HG002 (15Kb), HG002 (20Kb), HG002 (24Kb), CHM13 (20Kb), and SD0651_P1 (15Kb) (Supplementary Table 2). Details of the applied computing resources for the processes are shown in Supplementary Table 20. The runtime of the processes is shown in Supplementary Fig. 23. Note that for the first 3 processes, the runtime for each SMRT cell is shown. For the last 5 processes, the runtime for each "sample" (2 SMRT cells) is shown. The evaluation indicates that for a human sample with 2 SMRT cells of CCS reads, methylation phasing and ASM detection can be performed in less than 14 hours using ccsmethphase even on a single server (Supplementary Fig. 23).

Supplementary References

1 Robinson, J. T. *et al.* Integrative genomics viewer. *Nature Biotechnology* **29**, 24-26, doi:10.1038/nbt.1754 (2011).

2 Tse, O. O. *et al.* Genome-wide detection of cytosine methylation by single molecule real-time sequencing. *Proceedings of the National Academy of Sciences* **118** (2021).

3 Human Pangenome Reference Consortium. *HG002_Data_Freeze_v1.0*. (Human Pangenome Reference Consortium, accessed October 2022) https://github.com/human-pangenomics/HG002_Data_Freeze_v1.0.

4 Baid, G. *et al.* DeepConsensus improves the accuracy of sequences with a gap-aware sequence transformer. *Nature Biotechnology* (2022).

5 Nurk, S. *et al.* The complete sequence of a human genome. *Science* **376**, 44-53 (2022).

6 Oxford Nanopore Technologies. *ONT Open Datasets*. (Oxford Nanopore Technologies, accessed October 2022) https://labs.epi2me.io/dataindex/.

7 Zook, J. M. *et al.* Extensive sequencing of seven human genomes to characterize benchmark reference materials. *Scientific Data* **3**, 160025 (2016).

8 Liu, Y. *et al.* DNA methylation-calling tools for Oxford Nanopore sequencing: a survey and human epigenome-wide evaluation. *Genome Biology* **22**, 295 (2021).

9 Krueger, F. & Andrews, S. SNPsplit: Allele-specific splitting of alignments between genomes with known SNP genotypes [version 2; peer review: 3 approved]. *F1000Research* **5** (2016).

10 Li, H. Aligning sequence reads, clone sequences and assembly contigs with BWA-MEM. *arXiv preprint arXiv:1303.3997* (2013).

11 Kolesnikov, A. *et al.* DeepTrio: Variant Calling in Families Using Deep Learning. *bioRxiv*, 2021.2004.2005.438434 (2021).

12 Akbari, V. *et al.* Megabase-scale methylation phasing using nanopore long reads and NanoMethPhase. *Genome Biology* **22**, 68 (2021).

13 Krueger, F. & Andrews, S. R. Bismark: a flexible aligner and methylation caller for Bisulfite-Seq applications. *Bioinformatics* **27**, 1571-1572 (2011).

14 Park, Y. & Wu, H. Differential methylation analysis for BS-seq data under general experimental design. *Bioinformatics* **32**, 1446-1453 (2016).

15 Stoiber, M. *et al.* De novo Identification of DNA Modifications Enabled by Genome-Guided Nanopore Signal Processing. *bioRxiv*, 094672 (2017).

16 Ni, P. *et al.* DeepSignal: detecting DNA methylation state from Nanopore sequencing reads using deep-learning. *Bioinformatics* **35**, 4586-4595 (2019).

17 Zheng, Z. *et al.* Symphonizing pileup and full-alignment for deep learning-based long-read variant calling. *bioRxiv*, 2021.2012.2029.474431 (2021).

18 Martin, M. *et al.* WhatsHap: fast and accurate read-based phasing. *bioRxiv*, 085050 (2016).

19 Chung, J., Gulcehre, C., Cho, K. & Bengio, Y. Empirical evaluation of gated recurrent neural networks on sequence modeling. *arXiv preprint arXiv:1412.3555* (2014).

20 Bahdanau, D., Cho, K. & Bengio, Y. Neural machine translation by jointly learning to align and translate. *arXiv preprint arXiv:1409.0473* (2014).
