# Supplementary material for: DNA 5-methylcytosine detection and methylation phasing using PacBio circular consensus sequencing
Source: Nat Commun. 2023 Jul 8;14:4054. doi: 10.1038/s41467-023-39784-9 (PMC10329642; doi:10.1038/s41467-023-39784-9)
Supplement: Supplementary file 3 — Description of Additional Supplementary Files [file 41467_2023_39784_MOESM3_ESM.pdf]

Description of additional supplementary files

File Name: **Supplementary Data 1**

Description: Comparing ccsmeth and primrose/pb-CpG-tools against BS-seq and nanopore sequencing under different coverages of HG002 CCS reads (71.0× in total). Values for coverage 5×-70× are the average and standard deviation of 5 repeated tests in "average ± std" format. r: Pearson correlation; r^2: the coefficient of determination; ρ: Spearman correlation; RMSE: root mean square error.
