# Supplementary material for: DNA 5-methylcytosine detection and methylation phasing using PacBio circular consensus sequencing
Source: Nat Commun. 2023 Jul 8;14:4054. doi: 10.1038/s41467-023-39784-9 (PMC10329642; doi:10.1038/s41467-023-39784-9)
Supplement: Supplementary file 5 — Reporting Summary [file 41467_2023_39784_MOESM5_ESM.pdf]

nature portfolio

Corresponding author(s): Chuan-Le Xiao, Feng Luo, Jianxin Wang

Last updated by author(s): May 27, 2023

Reporting Summary

Nature Portfolio wishes to improve the reproducibility of the work that we publish. This form provides structure for consistency and transparency in reporting. For further information on Nature Portfolio policies, see our Editorial Policies and the Editorial Policy Checklist.

Statistics

For all statistical analyses, confirm that the following items are present in the figure legend, table legend, main text, or Methods section.

n/a	Confirmed	
☐	☒	The exact sample size (*n*) for each experimental group/condition, given as a discrete number and unit of measurement
☐	☒	A statement on whether measurements were taken from distinct samples or whether the same sample was measured repeatedly
☐	☒	The statistical test(s) used AND whether they are one- or two-sided<br>*Only common tests should be described solely by name; describe more complex techniques in the Methods section.*
☒	☐	A description of all covariates tested
☒	☐	A description of any assumptions or corrections, such as tests of normality and adjustment for multiple comparisons
☐	☒	A full description of the statistical parameters including central tendency (e.g. means) or other basic estimates (e.g. regression coefficient) AND variation (e.g. standard deviation) or associated estimates of uncertainty (e.g. confidence intervals)
☐	☒	For null hypothesis testing, the test statistic (e.g. *F*, *t*, *r*) with confidence intervals, effect sizes, degrees of freedom and *P* value noted<br>*Give P values as exact values whenever suitable.*
☒	☐	For Bayesian analysis, information on the choice of priors and Markov chain Monte Carlo settings
☒	☐	For hierarchical and complex designs, identification of the appropriate level for tests and full reporting of outcomes
☐	☒	Estimates of effect sizes (e.g. Cohen's *d*, Pearson's *r*), indicating how they were calculated

Our web collection on statistics for biologists contains articles on many of the points above.

Software and code

Policy information about availability of computer code

Data collection	Raw PacBio sequencing reads were sequenced on a Sequel II sequencer and processed with pbccs (v6.4.0, https://github.com/PacificBiosciences/ccs).
Data analysis	This manuscripts utilized open software and our scripts described in the Methods section and Supplementary Notes. We used pbccs (v6.4.0), ccsmeth (v0.3.2), primrose (version 1.3.0), pb-CpG-tools (v1.1.0), pbmm2 (v1.9.0), Clair3 (v0.1-r11 minor 2), WhatsHap (version 1.4), DSS (version 2.44.0), Nextflow (version 22.04.5.5708), Python3, PyTorch (version 1.11.0), Bismark (v0.23.1), Guppy (version 4.2.2+effbaf8), DeepSignal2 (v0.1.2), SNPsplit (version 0.5.0), DeepTrio (version 1.3.0), BWA-MEM (version 0.7.17-r1194-dirty), Tombo (version 1.5.1), UCSC Genome Browser (T2T CHM13v2.0/hs1), UCSC LiftOver, rasusa (v0.7.0), ccsmeth (https://github.com/PengNi/ccsmeth), ccsmethphase (https://github.com/PengNi/ccsmethphase). We also used HK model which was taken from Tse et al. (https://doi.org/10.1073/pnas.2019768118) under CUHK software license.

For manuscripts utilizing custom algorithms or software that are central to the research but not yet described in published literature, software must be made available to editors and reviewers. We strongly encourage code deposition in a community repository (e.g. GitHub). See the Nature Portfolio guidelines for submitting code & software for further information.

Data

Policy information about availability of data

All manuscripts must include a data availability statement. This statement should provide the following information, where applicable:

- Accession codes, unique identifiers, or web links for publicly available datasets
- A description of any restrictions on data availability
- For clinical datasets or third party data, please ensure that the statement adheres to our policy

This study is compliant with the “Guidance of the Ministry of Science and Technology (MOST) of China for the Review and Approval of Human Genetic Resources”. All sequencing data generated in this study have been deposited in the Genome Sequence Archive in National Genomics Data Center, Beijing Institute of Genomics (BIG, http://gsa.big.ac.cn), Chinese Academy of Sciences, under Project accession No. PRJCA015556[https://ngdc.cncb.ac.cn/bioproject/browse/PRJCA015556]. The sequencing data of NA12898 (GSA-Human accession No. HRA004180[https://ngdc.cncb.ac.cn/gsa-human/browse/HRA004180]) and the zebrafish sample (GSA accession No. CRA010412[https://ngdc.cncb.ac.cn/gsa/browse/CRA010412]) is available under open access. The sequencing data of SD0651_P1 and the HN0641 family trio (GSA-human accession No. HRA004202[https://ngdc.cncb.ac.cn/gsa-human/browse/HRA004202]) is available under restricted access, which can be granted by the Data Access Committee (DAC). Access can be obtained for Research Use Only by completing the application form via GSA. Users can register and login to GSA [https://ngdc.cncb.ac.cn/gsa-human/] and follow the guidance of “Request Data” [https://ngdc.cncb.ac.cn/gsa-human/document/GSA-Human_Request_Guide_for_Users_us.pdf] to request the data. The CCS datasets of M.SssI-treated and PCR-treated DNA (M01-03, W01-03) are available from Tse et al.(https://doi.org/10.1073/pnas.2019768118). The CCS reads of HG002 are available from Google Cloud [https://console.cloud.google.com/storage/browser/brain-genomics-public/research/deepconsensus/publication/sequencing] and the Human Reference Pangenome Consortium GitHub repository [https://github.com/human-pangenomics/HG002_Data_Freeze_v1.0]. Raw nanopore reads of HG002 are available at ONT Open Datasets [https://labs.epi2me.io/gm24385_2020.11/] with the flowcell ID PAG07165. The BS-seq reads of HG002 are also available at ONT Open Datasets [https://labs.epi2me.io/gm24385-5mc/]. The Illumina WGS 2×250bp reads of AshkenazimTrio (HG002, HG003, and HG004) are available at the GIAB GitHub repository [https://github.com/genome-in-a-bottle/giab_data_indexes]. The CHM13 CCS and nanopore reads are available at GitHub repository marbl/CHM1327[https://github.com/marbl/CHM13].

Human research participants

Policy information about studies involving human research participants and Sex and Gender in Research.

Reporting on sex and gender	Gender information was not collected as part of this study.
Population characteristics	The human male samples SD0651_P1, and three samples in a family trio (HN0641_FA male, HD0641_MO female, HN0641_S1 male) were selected from the Chinese autism spectrum disorder cohort. Among the four samples, SD0651_P1 was diagnosed as ASD. Population characteristics such as age, past and current diagnosis are not relevant to this study, as we only used the sequencing data of the human samples to validate the performance/accuracy of our methods for methylation detection and phasing.
Recruitment	The four human samples (SD0651_P1, HN0641_FA, HD0641_MO, HN0641_S1) were selected from the Chinese autism spectrum disorder cohort with no specific sex or age requirements. No selection bias would impact the results, as we only used the sequencing data of the human samples to validate the performance/accuracy of our methods for methylation detection and phasing.
Ethics oversight	The sequencing of the four human samples was approved by the Research Ethics Committee in in School of Life Sciences, Central South University (No. 2021-1-6).

Note that full information on the approval of the study protocol must also be provided in the manuscript.

Field-specific reporting

Please select the one below that is the best fit for your research. If you are not sure, read the appropriate sections before making your selection.

☒ Life sciences ☐ Behavioural & social sciences ☐ Ecological, evolutionary & environmental sciences

For a reference copy of the document with all sections, see nature.com/documents/nr-reporting-summary-flat.pdf

Life sciences study design

All studies must disclose on these points even when the disclosure is negative.

Sample size	Sample sizes were not predetermined. We have sequenced one SMRT cell CCS reads of NA19898, two SMRT cells CCS reads for each of the four human samples (SD0651_P1, HN0641_FA, HN0641_MO, HN0641_S1). We performed bisulfite sequencing of the SD0651_P1 sample. We also used publicly available CCS, ONT, BS-seq, WGS Illumina data of other human samples (M01, M02, M03, W01, W02, W03, HG002, CHM13). The number of individuals and the related sequencing data are sufficient to train and evaluate our proposed method and other tools.
Data exclusions	CCS reads that have less than 3 full-length subreads, and "Fail" Nanopore sequencing reads (mean Q-score <= 9) were not used in this study.
Replication	Results were evaluated across the full genome for every available human sample not used in model training. Especially, we performed

	orthogonal validation using PacBio CCS sequencing, ONT sequencing and/or bisulfite sequencing for HG002, CHM13, SD0651_P1. We also compared the results of sequencing data from different HG002 SMRT cells to validate the reproducibility. We listed the datasets used for model training and testing in Supplementary Tables 1-2. All the related data ensure the reproducibility of the results of our method.
Randomization	Sample randomization was not relevant to this study. The machine learning training followed standard practices for separating training, validation, and testing datasets. And we evaluated our method on sequencing data of each sample independently.
Blinding	Blinding was not relevant to this study. The machine learning training followed standard practices for separating training, validation, and testing datasets. And we evaluated our method on sequencing data of each sample independently.

Reporting for specific materials, systems and methods

We require information from authors about some types of materials, experimental systems and methods used in many studies. Here, indicate whether each material, system or method listed is relevant to your study. If you are not sure if a list item applies to your research, read the appropriate section before selecting a response.

Materials & experimental systems

n/a	Involved in the study
☒	☐ Antibodies
☐	☒ Eukaryotic cell lines
☒	☐ Palaeontology and archaeology
☐	☒ Animals and other organisms
☒	☐ Clinical data
☒	☐ Dual use research of concern

Methods

n/a	Involved in the study
☒	☐ ChIP-seq
☒	☐ Flow cytometry
☒	☐ MRI-based neuroimaging

Eukaryotic cell lines

Policy information about cell lines and Sex and Gender in Research

Cell line source(s)	NA12898/GM12898 cell line from the Coriell Institute
Authentication	No authentication was performed on cell lines
Mycoplasma contamination	The cell lines were not tested for Mycoplasma contamination. However, only the germline DNA content of the cell line are required, not any transcriptional or other cell phenotype.
Commonly misidentified lines (See ICLAC register)	No commonly misidentified lines were used.

Animals and other research organisms

Policy information about studies involving animals; ARRIVE guidelines recommended for reporting animal research, and Sex and Gender in Research

Laboratory animals	Danio rerio, TU wild-type line
Wild animals	N/A
Reporting on sex	male and female. Sex is not relevant to this study, as we only used the sequencing data of the zebrafish sample to validate the performance/accuracy of our methods for methylation detection.
Field-collected samples	N/A
Ethics oversight	The zebrafish adults were raised and provided according to established protocols by China Zebrafish Resource Center.

Note that full information on the approval of the study protocol must also be provided in the manuscript.
